# A Systematic Scoping Review on Portfolios of Medical Educators

**DOI:** 10.1177/23821205211000356

**Published:** 2021-03-24

**Authors:** Daniel Zhihao Hong, Annabelle Jia Sing Lim, Rei Tan, Yun Ting Ong, Anushka Pisupati, Eleanor Jia Xin Chong, Chrystie Wan Ning Quek, Jia Yin Lim, Jacquelin Jia Qi Ting, Min Chiam, Annelissa Mien Chew Chin, Alexia Sze Inn Lee, Limin Wijaya, Sandy Cook, Lalit Kumar Radha Krishna

**Affiliations:** 1 Yong Loo Lin School of Medicine, National University of Singapore, Singapore; 2 Division of Supportive and Palliative Care, National Cancer Centre Singapore, Singapore; 3 Division of Cancer Education, National Cancer Centre Singapore, Singapore; 4 Medical Library, National University of Singapore Libraries, Singapore; 5 Duke-NUS Medical School, Singapore; 6 Division of Infectious Disease, Singapore General Hospital, Singapore; 7 Palliative Care Institute Liverpool, Academic Palliative & End of Life Care Centre, Cancer Research Centre, University of Liverpool, UK; 8 Centre of Biomedical Ethics, National University of Singapore, Singapore; 9 PalC, The Palliative Care Centre for Excellence in Research and Education, Singapore; 10 Palliative Care Institute Liverpool, Academic Palliative & End of Life Care Centre, University of Liverpool, United Kingdom

**Keywords:** Medical educator portfolio, teaching portfolio, medical education, teaching, assessment, reflection

## Abstract

**Background:**

Heralded as a teaching, assessment and reflective tool, and increasingly as a longitudinal and holistic perspective of the educator’s development, medical educator’s portfolios (MEP)s are increasingly employed to evaluate progress, assess for promotions and career switches, used as a reflective tool and as a means of curating educational activities. However, despite its blossoming role, there is significant dissonance in the content and structure of MEPs. As such, a systematic scoping review (SSR) is proposed to identify what is known of MEPs and its contents.

**Methods:**

Krishna’s Systematic Evidenced Based Approach (SEBA) was adopted to structure this SSR in SEBA of MEPs. SEBA’s constructivist approach and relativist lens allow data from a variety of sources to be considered to paint a holistic picture of available information on MEPs.

**Results:**

From the 12 360 abstracts reviewed, 768 full text articles were evaluated, and 79 articles were included. Concurrent thematic and content analysis revealed similar themes and categories including: (1) *Definition and Functions of MEPs*, (2) *Implementing and Assessing MEPs*, (3) *Strengths and limitations of MEPs* and (4) *electronic MEPs*.

**Discussion:**

This SSR in SEBA proffers a novel 5-staged evidence-based approach to constructing MEPs which allows for consistent application and assessment of MEPs. This 5-stage approach pivots on assessing and verifying the achievement of developmental milestones or ‘micro-competencies’ that facilitate micro-credentialling and effective evaluation of a medical educator’s development and entrust-ability. This allows MEPs to be used as a reflective and collaborative tool and a basis for career planning.

## Introduction

Portfolios provide a holistic and longitudinal self-portrait of a medical educator’s professional identity formation and career development.^1-3^ Using self-selected material and reflections, portfolios^3-7^ differ sharply from logbooks, curriculum vitae, course logs, and training folders as a better means of evaluating a professional holistically and longitudinally.^1,3,4,8,9^ These self-portraits have even been used by medical educators as a means of illustrating their many roles^10-15^ for employment and promotion purposes.^16-18^ Indeed, medical educator portfolios (henceforth MEPs) circumnavigate the limitations posed by conventional assessment methods that often focus upon research grants and publications^5-7,15,18-20^ and to the detriment of appreciating the quality, breadth, depth^19,21^, and impact^11^ of a medical educator’s role amongst other things a *‘Professional Expert’, ‘Facilitator’, ‘Information Provider’, ‘Enthusiast’, ‘Faculty Developer’, ‘Mentor’, ‘Undergraduate and Postgraduate Trainer’, ‘Curriculum Developer’, ‘Assessor and Assessment Creator’, ‘Influencer’, ‘Scholar’, ‘Innovator’, ‘Leader’ and ‘Researcher’*.^13^

Increasing use of electronic portfolios ^22^ have further boosted the visibility of MEPs,^2,3,6,14,16-18,23,24^ and expanded its use in collaborative work and mentoring, making MEPs a valuable tool to assess medical educators,^1,17^ and underlining its increasing footprint in the medical education landscape.^1,4,25^

However, despite its much heralded benefits,^2,3,6,14,16-18,23,24^ various considerations in MEP’s structure, implementation, and assessments challenge its validity^2,7,14,18,21^. A systematic scoping review (SSR) is proposed to study current literature to enhance understanding of MEPs, its roles, its structure, and help to design a consistent framework for MEPs that can be used across settings, purposes, and specialities, given its ability to evaluate data^26-30^ from ‘*various methodological and epistemological traditions*’.^31^

## Methodology

To overcome a lack of structuring and the reflexive nature of SSRs, which raises questions to their reproducibility and transparency, we adopt Krishna’s Systematic Evidenced Based Approach (henceforth SEBA)^32-35^ to guide the SSR (henceforth SSR in SEBA) of MEPs. SSRs in SEBA proffer accountable, transparent and reproducible reviews.

To enhance accountability and transparency, SSRs in SEBA employ an expert team to guide, oversee, and support all stages of SEBA. The expert team is composed of composed of medical librarians from the Yong Loo Lin School of Medicine (YLLSoM) at the National University of Singapore (NUS) and the National Cancer Centre Singapore (NCCS), and local educational experts and clinicians at NCCS, the Palliative Care Institute Liverpool, YLLSoM and Duke-NUS Medical School. The expert team were involved in all stages of the SSR in SEBA.

SSRs in SEBA are built on a constructivist perspective. It acknowledges the personalised, reflective and experiential aspect of development as a medical educator, as well as medical education as a sociocultural construct influenced by prevailing clinical, academic, personal, research, professional, ethical, psychosocial, emotional, legal, and educational factors.^36-40^ This enables them to map data on a specific topic from multiple angles and consider the factors influencing the adoption of MEPs.

**Figure 1.**
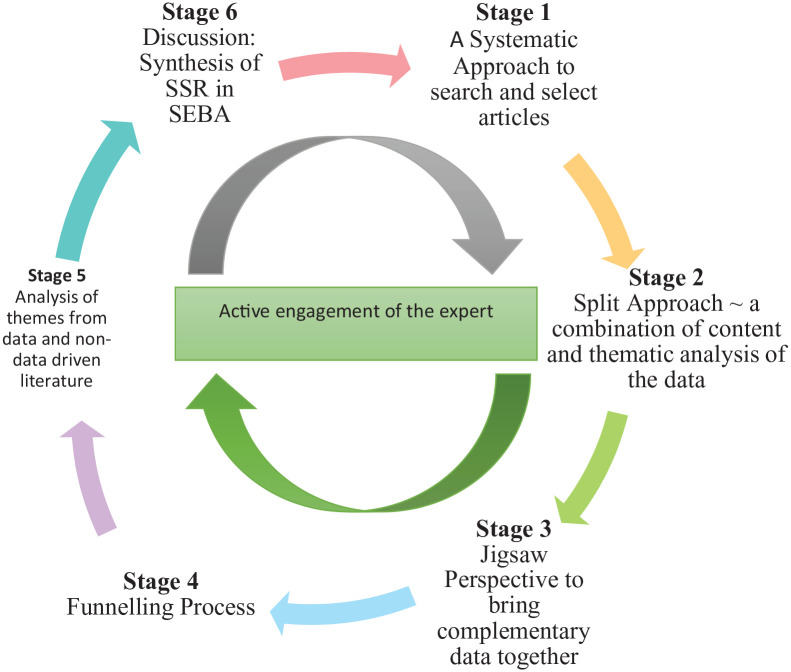
The SEBA process.

To operationalise a SSR in SEBA, the research team adopted the principles of interpretivist analysis, to enhance reflexivity and discussions^30,41-43^ in the Systematic Approach, Split Approach,^44-47^ Jigsaw Perspective, Funnelling Process, analysis of data from grey and black literature, and Synthesis of SSR in SEBA which make up SEBA’s 6 stages outlined in Figure 1.

### Stage 1 of SEBA: Systematic approach

#### Determining the title and background of the review

The expert and research team worked together to determine the overall goals of the SSR and the population, context, and concept to be evaluated. With increasing focus on the evaluation of educational activities amongst clinical faculties,^48^ it was deemed reasonable for MEPs to focus exclusively on educational activity and be distinct from a clinical portfolio, given prevailing suggestions that clinical accomplishments and development tend to cloud educational achievements.^1^

#### Identifying the research question

Guided by the Population, Concept, and Context (PCC), the teams agreed upon the research questions. The primary research question was ‘*what is known about medical educator portfolios*?’. The secondary questions were ‘*what are its components*?’, ‘*how are MEPs implemented*?’ and ‘*what are the strengths and weaknesses of current MEPs*?’.

#### Inclusion criteria

All grey literature, peer reviewed articles, narrative reviews, systematic, scoping, and systematic scoping reviews published from 1st January 2000 to 31st December 2019 were included in the PCC and a PICOS format was adopted to guide the research processes^49,50^ (See Supplemental File 1).

#### Searching

A search on 6 bibliographic databases (PubMed, Embase, PsycINFO, ERIC, Google Scholar and Scopus) was carried out between 17th of November 2019 to 24th of April 2020 for articles published between the years 2000 to 2019. Limiting the inclusion criteria to these dates was in keeping with Pham et al (2014)’s^51^ approach of ensuring a viable and sustainable research process. The search process adopted was structured along the processes set out by systematic scoping reviews. Additional articles were identified through snowballing.

The PubMed Search Strategy may be found in Supplemental File 2.

#### Extracting and charting

Using the abstract screening tool, members of the research team independently reviewed the titles and abstracts and created independent lists of titles to be reviewed. These lists were discussed online, and Sambunjak, Straus, Marusic’s^52^ approach to ‘negotiated consensual validation’ was used to achieve consensus on the final list of articles to be scrutinised.

The 6 members of the research team independently reviewed all articles on the final list, discussed them online, and adopted Sambunjak, Straus, Marusic’s^52^ approach to ‘negotiated consensual validation’ to achieve consensus on the final list of articles to be included.

### Stage 2 of SEBA: Split approach

Three teams of researchers simultaneously and independently reviewed the 79 included full-text articles. The first team of 3 researchers independently summarised and tabulated the included full-text articles in keeping with recommendations drawn from Wong, Greenhalgh, Westhorp, Buckingham, Pawson’s^53^ *RAMESES publication standards: meta-narrative reviews* and Popay, Roberts, Sowden, Petticrew, Arai, Rodgers, Britten, Roen, Duffy’s^54^ ‘*Guidance on the conduct of narrative synthesis in systematic reviews*’. These individual efforts were compared and discussed by the 3 researchers and consensus was achieved on the final content and structure of the tabulated summaries. The tabulated summaries served to ensure that key aspects of included articles were not lost.

Concurrently, the second team of 3 researchers independently analysed the included articles using Braun, Clarke’s^55^ approach to thematic analysis.^56^ In phase 1 of Braun and Clarke’s approach, the research team carried out independent reviews, ‘actively’ reading the included articles to find meaning and patterns in the data.^57-61^ In phase 2, ‘codes’ were constructed from the ‘surface’ meaning and collated into a code book to code and analyse the rest of the articles using an iterative step-by-step process. As new codes emerged, these were associated with previous codes and concepts. In phase 3, the categories were organised into themes that best depict the data. An inductive approach allowed themes to be ‘*defined from the raw data without any predetermined classification*’ .^60^ In phase 4, the themes were refined to best represent the whole data set and discussed. In phase 5, the research team discussed the results of their independent analysis online and at reviewer meetings. ‘*Negotiated consensual validation*’ was used to determine a final list of themes approach and ensure the final themes.^52^

A third team of 3 researchers independently analysed the included articles using Hsieh, Shannon’s^62^ approach to directed content analysis. Analysis using the directed content analysis approach involved ‘*identifying and operationalising a priori coding categories*’.^62-67^ The first stage saw the research team draw categories from Baldwin, Chandran, Gusic’s^68^ article entitled ‘*Guidelines for evaluating the educational performance of medical school faculty: priming a national conversation*’ to guide the coding of the articles. Any data not captured by these codes were assigned a new code. In keeping with deductive category application, coding categories were reviewed and revised as required.

In the third stage, the research team discussed their findings online and used ‘negotiated consensual validation’ to achieve consensus on the categories delineated and the codes within them. The final codes were compared and discussed with the final author, who checked the primary data sources to ensure that the codes made sense and were consistently employed. Any differences in coding were resolved between the research team and the final author. ‘Negotiated consensual validation’ was used as a means of peer debrief in all 3 teams to further enhance the validity of the findings.^69^

## Results

A total of 12 360 abstracts were reviewed, 768 full text articles were evaluated, and 79 articles were included (see Supplemental File 3). The themes identified using Braun, Clarke’s^55^ approach to thematic analysis and categories identified through use of Hsieh, Shannon’s^62^ approach to directed content analysis were similar and included (1) Definition and functions of MEPs, (2) Developing and implementing MEPs, (3) Assessing MEPs, (4) Strengths and limitations of MEPs and Electronic MEPs (E-MEP)s. See Table 1.

**Table 1.** Themes/categories by jigsaw perspective.

S/N	Themes/Categories by Jigsaw Perspective	Sub-themes
1	Definition and Functions of MEPs	Definition
		Functions of MEPs
2	Developing and implementing MEPs	Designing MEPs
		Components of MEPs
		Implementation of MEPs
3	Assessing MEPs	Formative and/or summative assessment of MEPs
		MEPs may contain quantitative and/or qualitative information.
		Setting standards/rubrics for assessment
		Assessors of portfolio
4	Strengths and Limitations of MEPs and Electronic MEPs (E-MEP)s	Strengths of MEPs
		Strengths of E-MEPs
		Limitations of MEPs
		Limitations of E-MEPs

### Stage 3 of SEBA: Jigsaw perspective

The Jigsaw Perspective sees the themes identified using Braun, Clarke’s^55^ approach to thematic analysis and categories identified through use of Hsieh, Shannon’s^62^ approach to directed content analysis reviewed by the research and expert teams as part of SEBA’s reiterative process. These discussions determined that there were significant overlaps and similarities between the themes and categories allowing them to be considered and presented in tandem.

### Theme/Category 1: Definition and functions of MEPs

#### Definition of MEPs

MEPs are defined as a collection of documents spanning a period of time^4,5,7,14,17,20,70,71^ seeking to demonstrate developing competencies,^1-4,8,17-20,72^ desirable character traits,^8^ learning,^4,8^ challenges and improvements made^3,8,14^ in the field of medical education. Curated by the individual, these documents reflect the medical educator’s perspective of their development^1-4,8,17-20,72^ and contains elements of feedback^73^ and reflection on good and bad experiences.^1-4,8^

#### Functions of MEPs

MEPs are used by medical educators to highlight professional development, documentation, learning activities, educational undertakings, reflections and career planning, while institutions employ MEPs for assessment purposes.

MEPs serve several functions and are used by medical educators and institutions differently. First, medical educators use MEPs to highlight professional development. They record their appraisals,^1,2,4,18,23^ revalidations,^1,3,4,23^ accreditations,^3,23,70,73^ and promotions^1,2,4,5,14,15,17-20,68,70,73,74^ in an MEP, and this can also be used for applying for specific roles within educational settings.^1,6,18^ Second, they serve as a form of documentation, where medical educators document their competencies^1-4,8,14,17-19,75^ certification of standards of professional performance^3,4^, illustrate accomplishments and educational activities,^1-4,7,17,18,25,70,71,76^ demonstrate desirable character traits,^1,8^ highlight leadership roles and successes,^1,8,17,19,68^ and showcase teamwork.^8^ Third, medical educators use MEPs as a learning tool to guide professional and personal improvements. They highlight experiences^8,24,76,77^ and reflections,^4,6,14,70,74^ capture feedback from learners, peers, mentors and supervisors,^4^ set learning objectives and guide work towards the achievement of learning objectives,^4,78^ and help to plan future lessons based on past experiences.^4,8^

On the other hand, institutions employ MEPs for assessment purposes. It serves as an assessment tool to facilitate hiring and promotion of medical educators by selection committee,^2,4-6,14,18,21,23,68^ and to evaluate medical educator’s performance and impact.^2,6,18,68^ Furthermore, MEPs help with the review of program accreditation.^79^

### Theme/Category 2: Developing and implementing MEPs

#### Designing MEPs

MEPs attempt to capture longitudinal development. Design of prevailing MEPs occur in a stepwise fashion beginning with an understanding of prevailing use of portfolio^16-18,73,76^ and the guiding principles behind these design structures,^16-18,73,74,76^ and its benefits and limitations.^8,70^ To contextualise MEPs to the particular setting, speciality, and the desired role,^3,4,8^ designers often consult intended users^77^ and experts.^68,73,76^

The dominant guiding principle for the design of the prototype is the need to balance structure^1,3,4,7,77^ and flexibility.^8,25,73^ Structure takes the form of including ‘critical’ domains to be curated^1^ and a consistent format is employed to ensure that practical^23^ and local institutional needs,^17,74^ as well as minimum standards of MEPs are met.^77^ Flexibility^8,25,73^ revolves around the contents of the MEP where aspects are sought to effectively capture inventiveness and learning^73^ and documentation and reflections.^3,4^ The prototype^4^ is then piloted and review from experts^68,73,76^ and feedback^2,3^ sought from a small group further refines its components^77^. The feedback and lessons learnt may be used to educate future users.^3^

#### Components of MEPs

A variety of domains are listed within current MEPs. These domains reflect the setting and the goals of the MEP. How these domains are selected and structured are often not described nor discussed and are thus curated in Table 2.

**Table 2.** Components of MEP.

Sub-themes	Elaboration and/or examples
General	Cover page^2,71,80,81^ List of Contents^2,71,79,80,82-84^ Identification^72,83,85-90^ Personal particulars and contact details^72,83,85-90^ Present rank and organisation affiliation^83,86-92^ Present role^71,93-97^ Personal statement^2,6,17,79,81,89,93,98,99^ Teaching philosophy^1,3,5,6,14,17,20,22,71,72,75,81,86-88,90,94-115^ What the educator considers essential constituents and attributes for successful teaching and learning^1,6,71,75,87,94-98,100,101,104-106,115,116^, experiences that shaped teaching style^6,75,86,87,96,101,104,105,109,115,116^, motivation in teaching^1,75,100,104,112^. beliefs and principles^71,86,87,100,104,112^ reflections on strengths, weaknesses, challenges, and growth in teaching over time^81,105,106,109,115,116^ enthusiasm in teaching^98^ comparing own teaching approach with other/newer approaches^1^ Goals^1,2,5,7,14,20,75-77,79,84,86-88,93-95,97,99-103,105-108,110-113,115^ short- and long-term goals^2,6,110^ may be recorded and ought to be regularly re-evaluated^1,2,75,105^ should consider areas of interest in medical education, future duties to be undertaken, potential obstacles for progression^1,20,89,94,101,105^ Should be specific, measurable, attainable, relevant and time bound (SMART)^1,75,108^ outline of duties and roles^2,85,91,97,103,113,114,117^ Listing of key contributions to medical education and major educational activities^81,85,95,98,108,109,117,118^
Teaching and scholarship	Documentation of teaching evidence illustrating topics taught^1-3,5,6,14,15,18-21,25,71,72,76-83,85-96,98-112,114,115,117-126^ Individual exemplars of techniques, approaches and outcomes^7,78^ Or general materials used in lectures, tutorials, workshops or courses^1,2,6,15,19,20,71,86,92,94,107,110,121,124^ Or online multimedia^5,18,20,71,92,94,107,124^ Teaching pedagogy and modalities^19,69,79,81-83,85,90,99,103,105,110,112,114,116,122,124^. This should highlight alignment with learning objectives and learner needs^7,69,116^ should consider the characteristics of the learner population^5^ should have an interactive element^69^ Use of creative and realistic teaching pedagogy^7,100,107,112,116,124^ Adoption of best practice when developing teaching content^7,69,80,85,98,108,117^ Evidence of use of the individual’s teaching pedagogy by fellow educators^81,100,124^ Learner numbers and profile^2,6,15,19,20,72,75,79-83,85-91,95,96,99,101,103,105-108,112,117,119-122,124-126^ Balance between quantity of learners and quality of teaching impact on learner^69,75,83^ Teaching location and hours^15,20,69,75,80-83,85-92,95,96,99,100,103,105-108,112,117-119,121,122,124-126^ Includes number of hours spent devising the activity as well as its actual execution^69,105^ Teaching impact^7,14,69,81,85,88,90,98,108,109^ Invitations to teach^95,98,100,107,118,125^ Multi-source feedback and ratings^1,2,6,8,15-17,20,69,73,75,85,86,89,91,92,94-96,98,100,101,106,107,109,110,120,124^ Learner grades and feedback^5,7,15,20,69,71,72,74,75,79,81,83,84,86,89,92,94-96,98-100,105-110,112,117,118,121,122,124-126^ Based on standardised assessments^2^ Comparing pre and post teaching^2,69^ Mentor or supervisor feedback^20,75,107,109,112^ Peer feedback^2,6,15,71,75,77,79,83,85,92,95,96,98,99,107,109,110,112,118,124-126^ Self-evaluation^1,2,71,75,98,106,110^ Comparing one’s performance with the standard^1^ Reflective entries^1,2,8,24,69,73,76,79-81,85,90,105,108,116,118^ Context, analysis and response (2, 57) Reflect on defining teaching experiences^8,76^ Reflect on insufficiencies, what was learnt and how to improve teaching^8,24,80,85,116,118^ Reflect on how to utilising feedback and outcomes to better teaching^7,69,80,118^ Aids goal setting^74^
Mentorship and advising^5,14,15,19,22,69,71,72,75,80-83,85-91,93-96,98-103,105,106,108,110-112,116-125^	Mentoring duties^80,82,83,85-91,93,96,100,103,108,117,118,121,124^ List and profiles of mentees^69,72,75,80,82,83,86-91,93,94,96,98-101,103,105,108,110,118-122,124^ Mentoring goals and pedagogies^85,88,108,117^ Mentee’s awards and scholarly products^69,75,80,83,85-87,89-91,93,94,96,98-100,103,105,108,110,117,118,122,124^ Reflective entries^85,98,103,108,112,117^
Educational research products	Publications details^1,6,7,15,20-22,69,71-73,75,82-84,86,87,89,91,94-101,103-110,112,117,118,122,124,126^ Presentations and invited conferences^7,15,18,20,21,69,71,73,75,82-84,86,87,89-91,95-97,99,100,103-105,107-110,112,117,118,124^ Details of delivery method and impact^7,69,82,83,87,89,91,96,105,108,117^ Books and Chapters^2,18,20,69,71,86,91,96,99,106,107,124,126^ Details of role, impact, and methodology^69,96^ Research projects^2,3,5,18,71,72,82,96,98^ Details of role and impact^96^ Development of educational tools and/or modules^15,20,69,71,75,88,104,105,108,121,125^ Details of effect on other institutions/curriculums^7,20,69,75,88,100,108,118^
	Use of modern technology^71^ Research or project or educational grants^5,15,20,69,71,72,75,82,83,88,90,91,94,96,97,99,100,103,105,117,122,124^ Details of grant value and impact on program development^15,69,82,90,94,96,105^ Role in grant (Principal or Co-Investigator)^69,82,90,94,96,105^
Leadership and Administration	Leadership roles^5,14,15,21,69,71,72,75,80-83,85-91,93-106,108-112,116-120,122-125^ Details of duties, position and impact^15,69,80,82,83,85-91,93,96,98,99,101,103,105,106,108,116-118,120,122^ Directs curriculum through establishing challenging targets, proper distribution of resources and assessing standard of teaching and learning^69,106,108,117,118^ Reflective entries^85,90,108,117,118^ Administrative roles^3,15,69,71,72,75,80,82,83,86,87,90-107,110,111,120,122-126^ Organising courses^69,97,107^ or assisting in curriculum development^69,96,97,99,107^ Member of institution committees^20,69,71,83,86,90,93,94,96,97,99,101,103,105,106,110,117,122,124^ Part of student-faculty associations^20,71^
Curriculum development	Curriculum development^1,5,6,15,20,21,69,71,75,79,80,82,83,85,86,89-91,93-106,108-110,112,116-119,121-126^ Role and contribution to curriculum development^69,80,82,83,85,96,97,100,101,104-106,108,112,117,118,121,122,124^ Brief description of curriculums created including its implementation process and learner profile^69,80,82,83,85,86,89-91,94,96-98,100,101,103-106,108,110,116-118,121,122,124^ Evidence of needs analysis of students^89,90,98,106,108^ Assessment of curriculum^86,89,90,94,96-99,101,103,105,108,116,117,124^ Adoption of curriculum by other institutes^122^ Reflective entries^85,98,105,108,117^
Assessment of learners	Learner assessment^1,15,19,69,75,80,85,89-91,94,95,99,101-103,105,108-110,117,118,121,122,125^ Role in assessment of learners^101,103,108^ Details of number of learners assessed and its importance of evaluation to the program^15,69,75,80,85,90,101,103,105,108,117,118^ Creating new assessment modalities^69,75,80,89-91,107,110,117,121^ Adoption of best practice^117,127^ Use of assessment tools to evaluate learners’ knowledge, skills, behaviours, and actions^69,90,101,103^ Special attention paid to reliability and validity of assessment modality^94^ Reflective entries^80,103,108,117,118^
Formal recognition	Teaching awards^1,2,5-7,17,20,69,71,72,75,81,82,85,86,89,91-101,103,104,106-108,110-112,115,117,118,120-122,124,125^ Description of selection criteria for^94,96,106,110,120,124^ Reference letters or letters of appreciation/support^2,6,7,71,75,81,92,96,98,100,103,110,120,124,126^
Professional development (training and certification)	Attendance at medical education conferences, meetings, courses, seminars, workshops, or modules^1,2,6,18,71,73,82-84,86,89,92,94-97,99,100,103,106,107,109,111,112,115,117,122,124^ Post-graduate degrees, programs or CME activities in medical education^1,3,71,82-84,92,94-97,103,106,109,111,112,117,121,122^

#### Implementation of MEPs

There are similarly poorly described steps in implementing current MEPs. Implementation of MEPs can be grouped under 4 themes – user training, assessor training, support and integration into existing practice. With user training, teaching sessions should be carried out prior to implementation of portfolio practice.^8,23^ This includes highlighting the purpose and benefits when introducing portfolios which may increase portfolio uptake, and providing samples, templates, flowcharts and assessment criteria to medical educators for better clarity on how to create and use portfolios.^7,8,17,68,73^ Trainers in these sessions should stress the documentation of activities before details are forgotten,^1^ highlight the use of portfolios as an active learning tool which allows for self-directed learning, the sharing of teaching philosophy and goals, introduce how one may interact with peers via dissemination of work,^17,77^ and explain how one should be discerning in the selection of evidence to include for reflection.^2^ Second, assessor training can enhance reliability as an assessment tool,^4^ help the institute’s promotion committee identify essential components of quality performance and train assessors to work with one another to evaluate and interpret a portfolio.^73^ Third, support should be provided through telephone calls or in person. Mentors, facilitators and tutors may facilitate reflection^2^ and help review the portfolio or go through portfolio assessment criteria before evaluation.^6,73,76^ Additionally, administrative support such as an information technology team are essential for successful implementation of MEPs to troubleshoot user problems.^23^ Lastly, integration into practice may be done in a longitudinal manner where users have to fill in the portfolio over time, and portfolios may be standalone or an adjunct to existing documentation methods like a curriculum vitae,^16,76^ and may also be part of summative assessments.^73^

### Theme/Category 3: Assessing MEPs

Table 3 summarises the key subthemes associated with assessments of MEPs including its use as a formative and summative tool, the type of evidence required for assessment, the development of assessment rubrics, and the assessors.

**Table 3.** Assessment of MEP.

Sub-themes	Elaboration and/or Examples
Formative and/or summative assessment of MEPs	Formative assessment^8,16,74^ Usually involve quality improvement^16^ Inclusion and analysis of feedback^74^ Summative assessment^3,8,16,74^ Provides a transparent assessment^8^ Ensures negative elements are also included^8,74^ Emphasises the learning process within professional development^74^
MEPs may contain quantitative and/or qualitative information.	Qualitative entries Aided by use of validated tools and frameworks^16,19^ Used to evaluate goals, personal statement and philosophy and the outcomes of their application^17^ Quantitative entries (student results, awards, teaching hours etc.)^17,19^ Allows the same assessment rubric to be used to ensure fairness and reproducibility^19^ Easily analysed^69^ Assess both qualitative and quantitative items^15,19,20,69,72,75^
Setting standards/rubrics for assessment	Benefits of establishing standards and rubrics for assessment Allow for academic recognition across institutions^19,21^ Ensure sufficient rigour to provide a platform for continuous development^19^ Ensures educators meet the standard of practice^7,19^ Empowers use of a summative portfolio in high-stake evaluations^69^ Easily utilised to determine fitness for promotion by committee members^19^
	This may be achieved by developing a novel scoring criteria^4^ or using existing standards or analysis tools^7,16^ Providing details regarding how each criterion is rated^19^ Addressing reliability issues by ensuring a fair and standardised assessment^4^ Encourages transparency and objectivity^74^ Can be utilised by different institutions^19^ Need for review and revision of assessment criteria Assessment rubric may be reviewed by educational experts to improve reliability^19^ Revision/updating of assessment rubrics after feedback and discussion by users^19,74^ Assessment should be tailored to each institution Rating system should be contextual and relevant to institutional needs^69^ Weightage of each component varies according to needs^19^ Agreed upon by own team of expert educators^19^
Assessors of portfolio	Institute^69,73,74^ Use of a team of assessors to ensure comprehensive assessment^74^ Can be coaches/mentors^74,77^ Longitudinal engagement improves validity of assessment^77^ Peer^1,2,6,73^ Self^74^ Needs to be trained^19^ Assessors need to be clear regarding rating system^19^

### Theme/Category 4: Strengths and limitations of MEPs and E-MEPs

Table 4 showcases the strengths and limitations of MEPs and electronic-MEPs.

**Table 4.** Strengths and limitations of MEPs and E-MEPs.

Strengths of MEPs	
Sub-themes	Elaboration and/or examples
Impact on medical educators	Motivates life-long self-learning^1-4,6,8,14,18,20,24,71,75,77,78^ Through repeating phases of reflection, preparation and execution of learning goals^1-3,5,8,24,77^ Flexibility in managing and selection of content in one’s own portfolio^8,78^ Identification of areas for improvement^1,3,8,14,24,77,79^ Developing self-awareness through reflection^2,3,14,79^ Facilitates the planning of activities to undertake in the future^1,14,79^ Strengthens good learning attributes^3,77^ Fosters self-confidence^1,24^ Develops an inquisitive mind^1^ Promotes problem-solving skills^71^ Promotes courage and stepping out of comfort zones^1^ Encourages teamwork^75^ Acquisition of competency^3,24,77^ Portfolios motivates educator to reach targeted objectives^2^ Expertise improvement^1^ Facilitates career advancements^1,3,71,75,77^ Fosters student-tutor relationships^25^ Ease of organising documents for regular assessments as documents are already gathered^6^
Impact on teaching	Improves quality of teaching^1-3,20,75,77^ By preparing teachings based on previous experiences^2,79^ By reflecting and being flexible during teachings^2,20^ Through multisource feedback from peers and learners regarding teaching practices^2,6,75,77^ Through collaboration with peers by sharing different perspectives, experiences and thoughts^77,79^
Impact on patient	Feedback improves patient care^1,3^ and patient safety^76^
Impact on institute	Efficient assessment^71^
Features of portfolio	Offers both qualitative and quantitative evidence^16,19,20,69,75^ Stimulates reflection^1-4,8,24,75^ Occurs throughout the process from the inclusion of meaningful evidence to the recognition of strengths and insufficiencies^8^ Evaluates teaching practices, goals and philosophy^8^ More comprehensive than other forms of documentation^1,6,18,19,21,69,77,80^ More accurate assessment of competence than curriculum vitae and/or letters of recommendation and/or standardised tests^1,18,19,21,69,80^ Better at illustrating an educator’s teaching techniques, efficacy, objectives and philosophy^1,19^ Allows evaluation of less successful activities^2^
Strengths of E-MEPs	
User perspective	Diversity of evidence such as Audio-visual recordings^1,3,4,71^, Graphics^3^, Web projects^3^, and Digital media^1,3,22,71^ may be included into MEPs Ease of accessibility, maintenance, and function^1-3,7,22,71^ Increases reflection^8,9^ Enhanced portability^1,2,4^ and instant access^9^ Easy to update^22,25^, retrieve peers’ work and provide feedback^9,71^ Readily backed-up^1,6^ More presentable as compared to paper-based portfolios^6^ Fosters collaboration and sharing of portfolio^1,4,7,25^ Provides privacy and security^3,25,71^ Greater learning drive^2,22^ More user-friendly^2,25,71^
Faculty perspective	Assessors and/or mentors can easily access user’s portfolio^7,9,22^ Allows administrators to evaluate portfolios regularly^9^
Limitations of MEPs and E-MEPs	
Sub-themes	Elaboration and/or Examples
User perspective	**MEPs:** Time and effort required^2,8,23,25,77,79^ User stress when deciding what content to include^16^ Lack of user motivation^8,74^ Lack of user control over portfolio components^2^ and variability in rigidity or flexibility^2^ Assessment orientated **E-MEPs:** Lack of technologic skill required to navigate online platform Unacquainted^3,8,9^ Lack of technical support^3,8^ Unable to find the time to learn how to use^3,8^ Security Hacking^3,8^
Faculty perspective	**MEPs:** Time and effort required^8,20^ Cost to assess 23 Unnecessary^18^ Inadequate as a stand-alone measure of performance^18^ Presence of other documentation modalities already in use^18^ Lack of reliability^2,19^ subjectivity is a concern due to variability of portfolio content^7,19,20^ Issues with plagiarism^8^ **E-MEPs:** Resources Lack of availability of computers in workplace^9^ Increase expenditure to provide technological support and training^9,25^ With high expectations for a visually pleasing and functionally impeccable design, creating an e-portfolio for medical educators can be challenging^8^

### Stage 4 of SEBA: Funnelling

Reviewing the themes/categories identified through the Jigsaw Process and comparing them with the tabulated summaries highlighted in Supplemental File 4 allows verification of the themes/categories and ensure that there is no additional data to be included. The themes/categories are then reviewed again by the expert team to determine if they may be funnelled into larger themes/categories that will form the basis of the discussion.

### Stage 5 of SEBA: Analysis of peer-reviewed and non-data driven literature

Evidenced based data from bibliographic databases (henceforth evidence-based publications) were separated from grey literature and opinion, perspectives, editorial, letters and non-data-based articles drawn from bibliographic databases (henceforth non-data driven) and thematically analysed to determine if non-data driven accounts had influenced the final synthesis of the discussions and conclusions.

The key themes identified from the peer-reviewed evidence-based publications and non-data driven publications were identical and included:

- Definition and functions of MEPs
- Developing and implementing MEPs
- Assessing MEPs
- Strengths and limitations of MEPs and E-MEPs

There was consensus that themes from non-data driven and the peer-reviewed evidence-based publications were similar and did not bias the analysis untowardly.

## Discussion

The narrative produced from consolidating the themes/categories/tabulated summaries was guided by the Best Evidence Medical Education (BEME) Collaboration guide and the STORIES (Structured approach to the Reporting In healthcare education of Evidence Synthesis) statement.

### Stage 6: Synthesis of the SSR in SEBA

In answering its primary and secondary research questions, this SSR in SEBA provides a number of key insights into the creation and employ of MEPs. To begin MEPs chronicles the professional, personal, research, academic, education and learning journey and development of a medical educator through self-selected data points, descriptions and reflections. Medical educators see MEPs as a means of advancing their careers, capturing their experiences and reflections and as a learning tool, whilst for institutions, MEPs provide a wider perspective of the medical educator and an additional source of data to evaluate an education program.

With evidence that they motivate lifelong learning, self-improvements, promote the acquisition of competency and career advancement, and benefit learners by improving quality of teaching, patient safety and care and program efficacy, MEPs are gradually gaining traction amongst medical educators and institutions. These developments underline the need better structure MEPs to facilitate its wider use.

**Figure 2.**
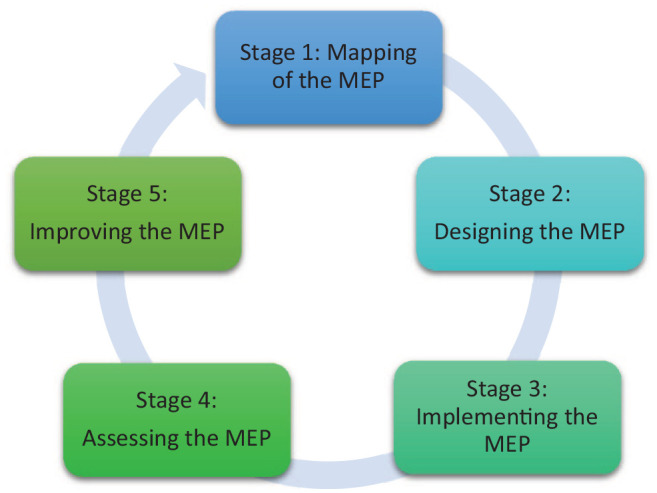
Five stages of construction and deployment of MEP.

Here we proffer a 5-staged evidence-based approach to the construction and deployment of a MEP as shown in Figure 2.

#### Stage 1: Mapping of the MEP

To begin with, a needs assessment should be carried out by the educational institute to determine, the need, goals,^17,74^ support and practical issues^9,25^ associated with implementing such a process. Party to this process must be an acceptable, transparent^16,19,20,68,74^ and verifiable^1,6,18,19,21,68,77,80^ means of evaluating the diverse contents of MEPs for accreditation and promotion.^16-18^ This takes the form of a purpose designed MEP.

#### Stage 2: Designing the MEP

To maximise its impact, a MEP must include longitudinal quantitative and qualitative evidence^15,19,20,68,71,74,75^ that is accompanied by clear documentation, reflections and be supplemented the medical educators’ many educational roles,^12,81-83^ competencies,^84-87^ characteristics, expectations^5,15,20,21,80^ and attainment of specific professional standards such as those set out by the Academy of Medical Educators (AoME)^84,88-92^ and the Accreditation Council for Graduate Medical Education (ACGME).^91,93-96^ Guiding this design are several considerations.

One, the competency based assessments of progression set out by the Academy of Medical Educators^84^ helps ensure that key elements of this assessment process is contained within the MEP. These competency based assessments of progression^84^ also align a medical educator’s learning objectives to the relevant competency guidelines, local context^7,68^ and an institution’s promotion criteria,^5,74^ making the MEP more applicable across settings^95,97^ and outcomes.^95,97^

Two, the need for a flexible framework that facilitates balance between flexibility to infuse personal data and the requisite for consistency to ensure that critical data is included. Only when a balance of structure and flexibility is obtained can the portfolio be an accurate depiction of the beliefs, attitudes, behaviours, and professional identity of the medical educator.^3^

Three, there must be adequate education of medical educators to ensure that they remain motivated to maintain this ‘living’ document and update it with their goals and plans for future career development. This will also foster effective use of the MEPs as a means of regular self-assessment, continuous education and reflection^3,95^ which will boost professional development.^3^

Four, for ease of review, access and personalisation, electronic MEPs ought to be employed. Electronic MEPs also allow application and storage of diverse evidence such as digital media and recordings and provide a convenient means of collaboration with peers and mentors.^1,4,7,9,25,98^ However, it is crucial to keep an electronic MEP user-friendly^2,25,98^ and well supported,^9^ to aid its adoption.^99^

Five, the combination of this template and use of an electronic platform facilitates adaption to local requirements^68^ and enable medical educators to personalise the MEP to their own needs, focuses, phases of their career^73^ and learning style.^3^

**Figure 3.**
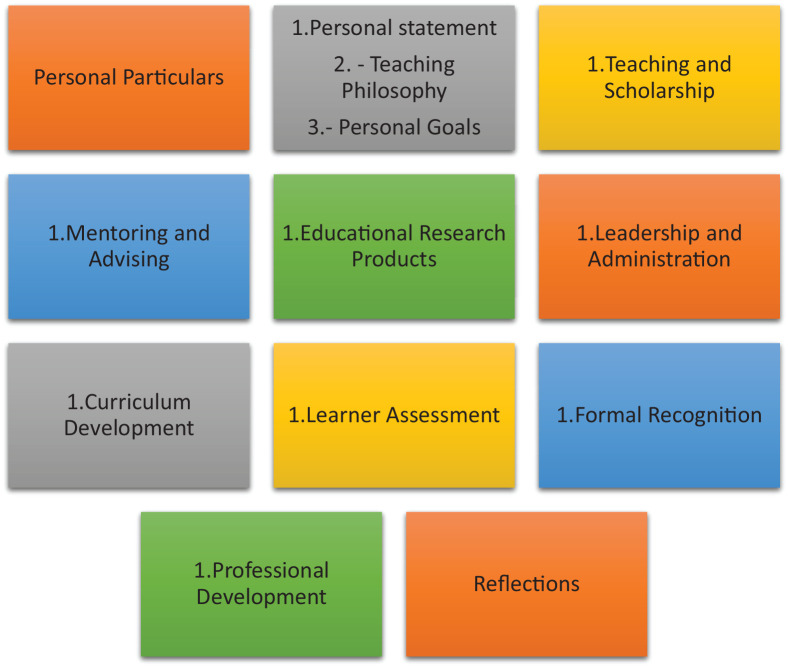
What should be documented in MEPs.

Based on these 5 considerations, we suggest that MEPs document these themes seen in Figure 3 below:

See also Supplemental File 5 for a MEP template based on these themes.

(Sections 3-10 should contain exemplars, innovations, evidence of progress/maturation of practice, evaluations, feedback and reflections and analysis of events, both positive and negative experiences)^100,101^

#### Stage 3: Implementing the MEP

Implementation of MEPs must be accompanied by the training of all users, assessors, and faculty as to the role, need, value and use of MEPs as well as how it is assessed.^70,102-105^ Exemplars^7,8^ and scoring rubrics,^19^ can guide new users^14,16^ and ensure fair assessments and improve reliability.^4,106^ To provide support and guidance for users, assessors and faculty,^99,102,104,107,108^ coaches, supervisors, and increasingly mentors^1^ should be made available to follow the learner’s progress.^99^ In turn these coaches, supervisors, and mentors must be provided with protected time^109^ and administrative support to help design, update and troubleshoot issues.^105^

#### Stage 4: Assessing the MEP

Pre-empting issues with assessing the various domains and diverse designs through use of qualitative, quantitative, and or mixed methods in the absence of a general standardised assessment rubric,^7^ local institutions could promote a homogenous portfolio structure which would aid in the creation of an assessment rubric^19^ and clear assessment criteria.^110^ Such a rubric may be drawn from Glassick’s criteria of educational excellence,^111-114^ Miller’s pyramid,^115^ the GNOME model of curriculum design,^116-118^ Kirkpatrick’s Model^119,120^ and Association of American Medical Colleges (AAMC) Toolbox.^121,122^ These tools will help overcome concerns about the lack of transparency and consistency in prevailing assessments of MEPs.^16,73,110,123-126^

### Stage 5: Updating and improving the MEP

As ‘living documents’ capturing the evolving self-concepts and professional and personal identities of medical educators’ and their changing goals, experiences, and MEPs need to be adapted, pared, and reviewed. Here the data suggests the presence of ‘micro-competencies’. ‘Micro-competencies’ are effectively milestones that are formally assessed and verified using multisource assessments contained within the portfolio. ‘Micro-competencies’ are evident in the developing medical educator’s entries within the portfolio. These entries replete with learning objectives, reports of training approaches and assessments used, the feedback garnered from these sessions, evidence of the longer-term impact upon the learners, the medical educator’s own reflections and plans for refinement provide evidence and verification to development.

‘Micro-competencies’ suggest that a medical educator’s skills, knowledge, and attitudes develop in stepwise competency-based stages from early medical training and continue till all the micro-competencies and competencies are met. These micro-competencies and competencies are then honed and refined by master medical educators. We see the use of these verified achievements of milestones as a natural progression of the concept of milestones within the context of MEPs. ‘Micro-competencies’ guide the medical educator’s development and inform appraisals of their progress, coping, conduct and development. Critically rather than merely standardized points to be met along the trajectory towards achieving a competency, micro-competencies within the MEPs allow a number of refinements to the traditional concept of milestones.

One, micro-competencies are variable and determined with due consideration of the medical educator’s abilities, skills, level of practice, experience, training, and clinical and or professional roles and responsibilities as well as their practice settings and sociocultural context. This highlights the personalised features of micro-competencies.

Two, when considered in tandem with established milestones expected of all medical educators, micro-competencies also highlight the ‘general’ aspect of micro-competencies. The general aspect of micro-competencies is drawn from ‘stage specific requirements’ that all medical educators should achieve at a specific stage of their training.

Three, micro-competencies also vary with setting, stages of training, context, and time. Changes in these aspects of practice require re-evaluation of the medical educator’s micro-competencies. Micro-competencies allow the tutors, supervisors, reviewers, mentors, coaches, supervisors, assessors (henceforth faculty) and or employers to evaluate progress and provide medical educators with an opportunity to re-evaluate and reflect on their development and focus upon developing their learning plan.

Four, micro-competencies also acknowledge that they may be the basis of more than one competency and that without regular application will result in degradation of their abilities specifically communication and skills based micro-competencies. This highlights the time-specific nature of the micro-competency. Similarly, with medical educators often posted to different settings and or participate in training in different specialties involving learners of different backgrounds, experience and training underline the need for timely re-evaluation of micro-competencies.

Overall use of MEPs evidences the notion that micro-credentialling^127^ could be built upon the achievement of personalised and general micro-competencies. Micro-credentialling allows medical educators, the organisation, the evaluators and potential recruiters to see the specific settings that a medical educator can function within, the capacity or roles and responsibilities that they can adopt, the level of supervision required and their overall progress towards attaining Entrustable Professional Activities (EPAs). With EPAs built on micro-credentials, the trajectory and gaps on the course towards attaining a specific EPA are mapped out, aiding medical educators as they reflect upon and map their course towards their overall goals. Progress captured in longitudinal assessments will also help medical educators and faculty to personalise training and support programs.

Overall micro-competencies, their relationship with micro-credentialling and EPAs inform guidance on personal, professional, and research expectations upon medical educators and steer effective career progression, maturation of thought, philosophies, skills, and actions.

## Limitations

Whilst our goal was to appreciate the scope of available literature on portfolios used by medical educators, this review is limited by the lack of longitudinal and holistic evaluations of portfolios.

Although the search process was vetted and overseen by the expert team, use of specific search terms and inclusion of only English language articles potentiates the possibility of key publications being omitted. In addition, whilst independent and concurrent use of thematic and content analysis by the team of researchers improved its trustworthiness through enhanced triangulation and transparency, biases cannot be entirely eradicated.

The inclusion of grey literature improves transparency in the synthesis of the discussion, but its themes may contain bias results and provide these opinion-based views with a ‘veneer of respectability’ despite a lack of evidence to support it. This raises the question as to whether grey literature should be accorded the same weight as published literature.

## Conclusions

This SSR in SEBA has laid bare the range of data on MEPs and highlighted the gaps in prevailing concepts. Perhaps a critical consideration is the fact that MEPs continue to be used for a variety of roles and goals and remain influenced by local clinical, academic, personal, research, professional, ethical, psychosocial, emotional, cultural, societal, legal and educational factors underlining the heterogeneity of available data.

Recognising this fact, we propose to determine the key ‘ingredients’ of successful MEPs in a coming study. In the meantime, we look forward to continuing this discussion, evaluating how best to ensure this living document is effectively tended to and how effective and appropriate training and assessment processes can be set up to realise the full potential of MEPs.

## Supplemental Material

sj-pdf-1-mde-10.1177_23821205211000356 – Supplemental material for A Systematic Scoping Review on Portfolios of Medical Educators Click here for additional data file. Supplemental material, sj-pdf-1-mde-10.1177_23821205211000356 for A Systematic Scoping Review on Portfolios of Medical Educators by Daniel Zhihao Hong, Annabelle Jia Sing Lim, Rei Tan, Yun Ting Ong, Anushka Pisupati, Eleanor Jia Xin Chong, Chrystie Wan Ning Quek, Jia Yin Lim, Jacquelin Jia Qi Ting, Min Chiam, Annelissa Mien Chew Chin, Alexia Sze Inn Lee, Limin Wijaya, Sandy Cook and Lalit Kumar Radha Krishna in Journal of Medical Education and Curricular Development

sj-pdf-2-mde-10.1177_23821205211000356 – Supplemental material for A Systematic Scoping Review on Portfolios of Medical Educators Click here for additional data file. Supplemental material, sj-pdf-2-mde-10.1177_23821205211000356 for A Systematic Scoping Review on Portfolios of Medical Educators by Daniel Zhihao Hong, Annabelle Jia Sing Lim, Rei Tan, Yun Ting Ong, Anushka Pisupati, Eleanor Jia Xin Chong, Chrystie Wan Ning Quek, Jia Yin Lim, Jacquelin Jia Qi Ting, Min Chiam, Annelissa Mien Chew Chin, Alexia Sze Inn Lee, Limin Wijaya, Sandy Cook and Lalit Kumar Radha Krishna in Journal of Medical Education and Curricular Development

sj-pdf-3-mde-10.1177_23821205211000356 – Supplemental material for A Systematic Scoping Review on Portfolios of Medical Educators Click here for additional data file. Supplemental material, sj-pdf-3-mde-10.1177_23821205211000356 for A Systematic Scoping Review on Portfolios of Medical Educators by Daniel Zhihao Hong, Annabelle Jia Sing Lim, Rei Tan, Yun Ting Ong, Anushka Pisupati, Eleanor Jia Xin Chong, Chrystie Wan Ning Quek, Jia Yin Lim, Jacquelin Jia Qi Ting, Min Chiam, Annelissa Mien Chew Chin, Alexia Sze Inn Lee, Limin Wijaya, Sandy Cook and Lalit Kumar Radha Krishna in Journal of Medical Education and Curricular Development

sj-pdf-4-mde-10.1177_23821205211000356 – Supplemental material for A Systematic Scoping Review on Portfolios of Medical Educators Click here for additional data file. Supplemental material, sj-pdf-4-mde-10.1177_23821205211000356 for A Systematic Scoping Review on Portfolios of Medical Educators by Daniel Zhihao Hong, Annabelle Jia Sing Lim, Rei Tan, Yun Ting Ong, Anushka Pisupati, Eleanor Jia Xin Chong, Chrystie Wan Ning Quek, Jia Yin Lim, Jacquelin Jia Qi Ting, Min Chiam, Annelissa Mien Chew Chin, Alexia Sze Inn Lee, Limin Wijaya, Sandy Cook and Lalit Kumar Radha Krishna in Journal of Medical Education and Curricular Development

sj-pdf-5-mde-10.1177_23821205211000356 – Supplemental material for A Systematic Scoping Review on Portfolios of Medical Educators Click here for additional data file. Supplemental material, sj-pdf-5-mde-10.1177_23821205211000356 for A Systematic Scoping Review on Portfolios of Medical Educators by Daniel Zhihao Hong, Annabelle Jia Sing Lim, Rei Tan, Yun Ting Ong, Anushka Pisupati, Eleanor Jia Xin Chong, Chrystie Wan Ning Quek, Jia Yin Lim, Jacquelin Jia Qi Ting, Min Chiam, Annelissa Mien Chew Chin, Alexia Sze Inn Lee, Limin Wijaya, Sandy Cook and Lalit Kumar Radha Krishna in Journal of Medical Education and Curricular Development

sj-pdf-6-mde-10.1177_23821205211000356 – Supplemental material for A Systematic Scoping Review on Portfolios of Medical Educators Click here for additional data file. Supplemental material, sj-pdf-6-mde-10.1177_23821205211000356 for A Systematic Scoping Review on Portfolios of Medical Educators by Daniel Zhihao Hong, Annabelle Jia Sing Lim, Rei Tan, Yun Ting Ong, Anushka Pisupati, Eleanor Jia Xin Chong, Chrystie Wan Ning Quek, Jia Yin Lim, Jacquelin Jia Qi Ting, Min Chiam, Annelissa Mien Chew Chin, Alexia Sze Inn Lee, Limin Wijaya, Sandy Cook and Lalit Kumar Radha Krishna in Journal of Medical Education and Curricular Development
