# Supplementary material for: A Systematic Scoping Review on Portfolios of Medical Educators
Source: J Med Educ Curric Dev. 2021 Mar 24;8:23821205211000356. doi: 10.1177/23821205211000356 (PMC8855455; doi:10.1177/23821205211000356)
Supplement: sj-pdf-1-mde-10.1177_23821205211000356 – Supplemental material for A Systematic Scoping Review on Portfolios of Medical Educators [file sj-pdf-1-mde-10.1177_23821205211000356.pdf]

Supplementary File 1. <u>PICOS, Inclusion and Exclusion Criteria for Medical Educator Portfolio Reviews</u>

<table>
<tr><td>PICOS</td><td>Inclusion criteria</td><td>Exclusion criteria</td></tr>
<tr><td>Population</td><td>Current or aspiring medical educators and/or faculty members within the clinical, medical, research and/or academic settings</td><td>Undergraduate and postgraduate medical students
Residents and physicians not involved in medical education
Allied health specialties such as Pharmacy, Dietetics, Chiropractic, Midwifery, Podiatry, Speech Therapy, Occupational and Physiotherapy
Non-medical specialties such as Clinical and Translational Science, Alternative and Traditional Medicine, Veterinary, Dentistry</td></tr>
<tr><td>Intervention</td><td>Papers that addressed the use of portfolios for current or aspiring medical educators and/or faculty members within the clinical, medical, research and/or academic settings
Criteria essential to be considered a portfolio:
A collection of evidence of learning across time
It must also include personal intellectual engagement with the content of the portfolio and associated learning
Interventions that meet the above criteria were included regardless of whether they referred to their interventions as portfolios
All types of portfolios were considered in this study.</td><td>Other documentation methods or learning tools that are:
Not a collection but rather a singular piece of work
Only from a single time point
Does not include personal intellectual engagement with the content and associated learning (for instance, curriculum vitae, logbooks and the use of personal digital assistants)</td></tr>
<tr><td>Comparison</td><td>Papers that addressed the following comparisons were also included:
Comparison of the various uses of portfolios in different teaching settings</td><td></td></tr>
</table>

	• Comparison of the effectiveness of portfolios in comparison to other interventions	
Outcome	Papers that measured the following outcomes were also included:<br><br>• Impact of the use of portfolios on current or aspiring medical educators and/or faculty members within the clinical, medical, research and/or academic settings<br>• Impact of the use of portfolios on teaching<br>• Impact of the use of portfolios on faculty	
Study design	• All study designs including:<br>o Mixed methods research, meta-analyses, systematic reviews, randomized controlled trials, cohort studies, case-control studies, cross-sectional studies, descriptive papers, grey literature, opinions, letters, commentaries and editorials<br>• Articles in English or translated to English<br>• Year of Publication: 2000 – 2019	

Supplementary File 2. <u>PubMed Search Strategy</u>

		**Mesh Terms**	**tiab**
**Population**	Medical Students OR Doctors	[1] “Physicians”[MeSH] OR “Students, Medical”[MeSH] OR “Clinical Clerkship”[MeSH] OR "Medicine"[Mesh] OR "Education, Medical"[Mesh]	[2] Physician[tiab] OR Physicians[tiab] OR resident[tiab] OR residents[tiab] OR residency[tiab] OR residencies[tiab] OR practice[tiab] OR practitioner[tiab] OR practitioners[tiab] OR doctor[tiab] OR doctors[tiab] OR houseman[tiab] OR housemanship[tiab] OR housemen[tiab] OR medical[tiab] OR clinical[tiab] OR pre-clinical[tiab] OR preclinical[tiab] OR clinician*[tiab] OR surgery[tiab] OR

		OR "Clinical Competence"[Mesh]	surgical[tiab] OR surgeon*[tiab] OR clerkship*[tiab] OR specialist*[tiab]-
**Context**			[3] ("Educational Measurement/methods"[Mesh] OR<br>“Educational Measurement/standards"[Mesh] OR<br>"Documentation/methods"[Mesh] OR “Benchmarking*”[MeSH] OR “Competency-based education/standards*”[MeSH] OR “Records*”[MeSH]) AND (medical[tiab] OR clinical[tiab] OR pre-clinical[tiab] OR preclinical[tiab] OR clinician*[tiab] OR surgery[tiab] OR surgical[tiab] OR surgeon*[tiab] OR clerkship*[tiab] OR specialist*[tiab])
**Intervention**	Portfolios		[4] Portfolio[tiab] OR portfolios[tiab] OR e-portfolio[tiab] OR e-portfolios[tiab] OR “curriculum vitae”[tiab] OR “personal statement”[tiab] OR “personal statements”[tiab]

(1 OR 2 OR 3) AND 4

Supplementary File 3. PRISMA Flowchart

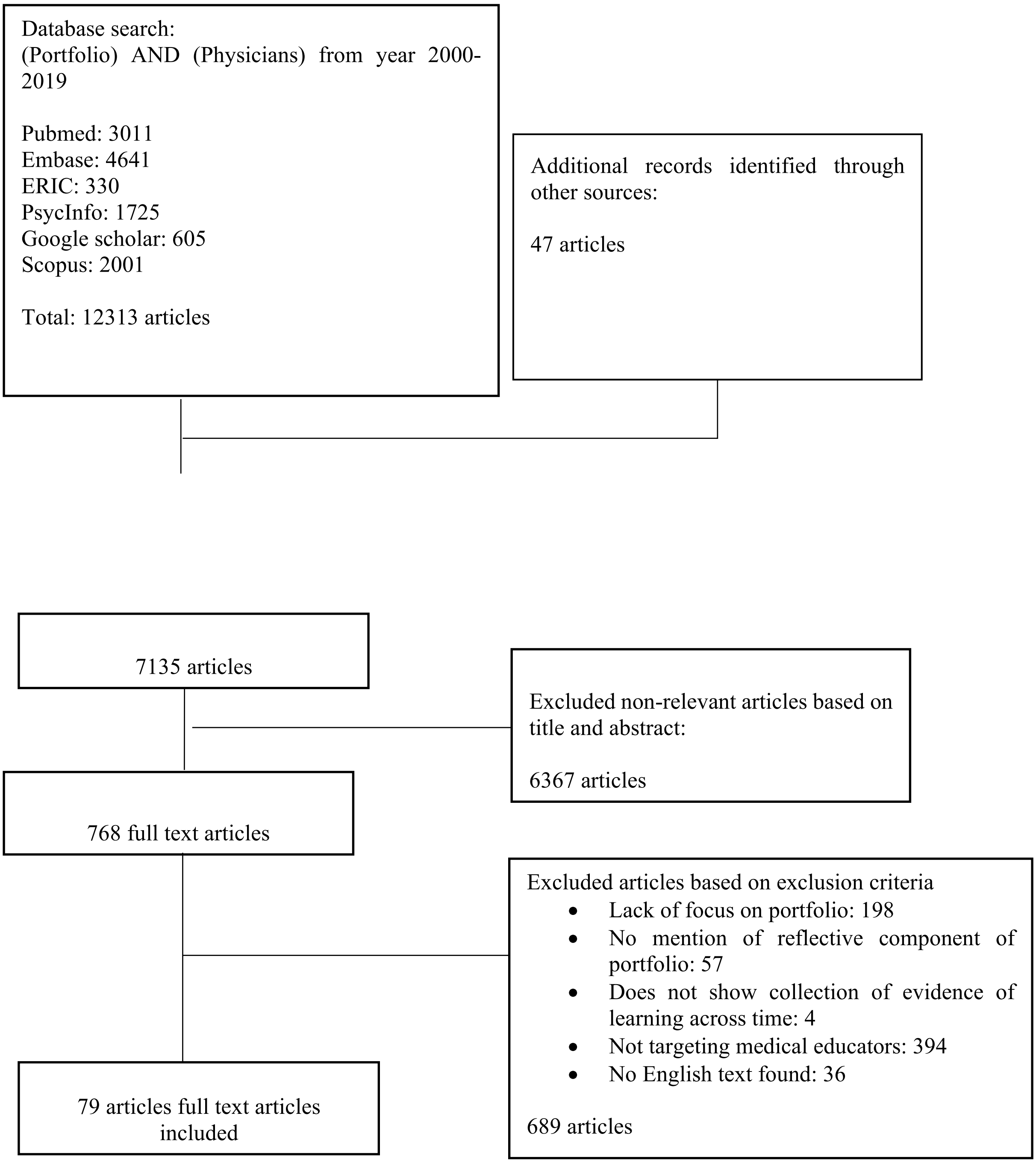
Database search:
(Portfolio) AND (Physicians) from year 2000-2019
Pubmed: 3011
Embase: 4641
ERIC: 330
PsycInfo: 1725
Google scholar: 605
Scopus: 2001
Total: 12313 articles
Additional records identified through other sources:
47 articles
7135 articles
Excluded non-relevant articles based on title and abstract:
6367 articles
768 full text articles
Excluded articles based on exclusion criteria
Lack of focus on portfolio: 198
No mention of reflective component of portfolio: 57
Does not show collection of evidence of learning across time: 4
Not targeting medical educators: 394
No English text found: 36
689 articles
79 articles full text articles included
