# Supplementary material for: A Systematic Scoping Review on Portfolios of Medical Educators
Source: J Med Educ Curric Dev. 2021 Mar 24;8:23821205211000356. doi: 10.1177/23821205211000356 (PMC8855455; doi:10.1177/23821205211000356)
Supplement: sj-pdf-2-mde-10.1177_23821205211000356 – Supplemental material for A Systematic Scoping Review on Portfolios of Medical Educators [file sj-pdf-2-mde-10.1177_23821205211000356.pdf]

Supplementary File 2. PubMed Search Strategy

		**Mesh Terms**	**tiab**
**Population**	Medical Students OR Doctors	[1] "Physicians"[MeSH] OR "Students, Medical"[MeSH] OR "Clinical Clerkship"[MeSH] OR "Medicine"[Mesh] OR "Education, Medical"[Mesh] OR "Clinical Competence"[Mesh]	[2] Physician[tiab] OR Physicians[tiab] OR resident[tiab] OR residents[tiab] OR residency[tiab] OR residencies[tiab] OR practice[tiab] OR practitioner[tiab] OR practitioners[tiab] OR doctor[tiab] OR doctors[tiab] OR houseman[tiab] OR housemanship[tiab] OR housemen[tiab] OR medical[tiab] OR clinical[tiab] OR pre-clinical[tiab] OR preclinical[tiab] OR clinician*[tiab] OR surgery[tiab] OR surgical[tiab] OR surgeon*[tiab] OR clerkship*[tiab] OR specialist*[tiab]-
**Context**			[3] ("Educational Measurement/methods"[Mesh] OR "Educational Measurement/standards"[Mesh] OR "Documentation/methods"[Mesh] OR "Benchmarking*"[MeSH] OR "Competency-based education/standards*"[MeSH] OR "Records*"[MeSH]) AND (medical[tiab] OR clinical[tiab] OR pre-clinical[tiab] OR preclinical[tiab] OR clinician*[tiab] OR surgery[tiab] OR surgical[tiab] OR surgeon*[tiab] OR clerkship*[tiab] OR specialist*[tiab])
**Intervention**	Portfolios		[4] Portfolio[tiab] OR portfolios[tiab] OR e-portfolio[tiab] OR e-portfolios[tiab] OR "curriculum vitae"[tiab] OR "personal statement"[tiab] OR "personal statements"[tiab]

(1 OR 2 OR 3) AND 4
