# Supplementary figures and images for: A Systematic Scoping Review on Portfolios of Medical Educators
Source: J Med Educ Curric Dev. 2021 Mar 24;8:23821205211000356. doi: 10.1177/23821205211000356 (PMC8855455; doi:10.1177/23821205211000356)

Supplementary File 3. *PRISMA Flowchart*

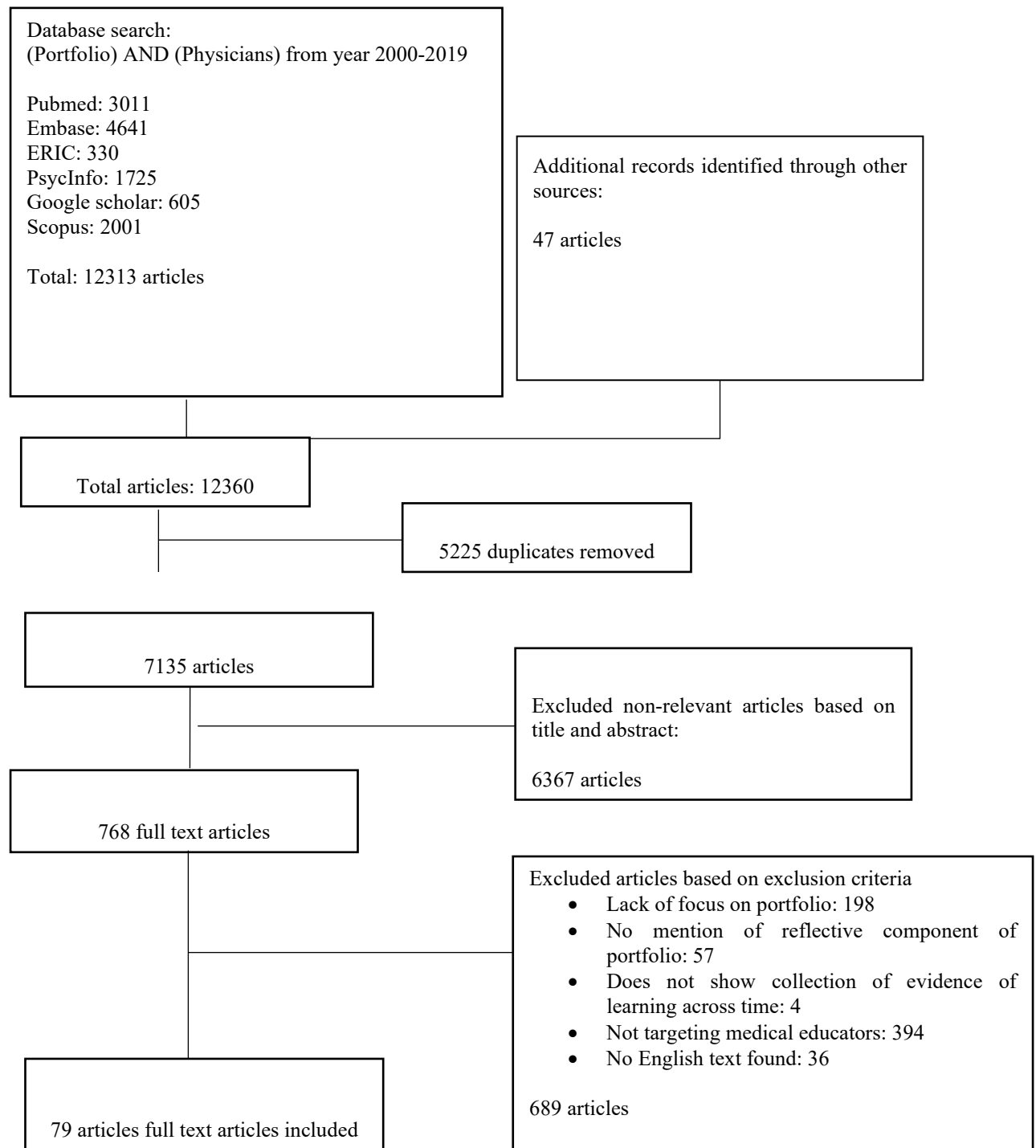

Supplement: sj-pdf-3-mde-10.1177_23821205211000356 – Supplemental material for A Systematic Scoping Review on Portfolios of Medical Educators [file sj-pdf-3-mde-10.1177_23821205211000356.pdf]
