# Supplementary material for: A Systematic Scoping Review on Portfolios of Medical Educators
Source: J Med Educ Curric Dev. 2021 Mar 24;8:23821205211000356. doi: 10.1177/23821205211000356 (PMC8855455; doi:10.1177/23821205211000356)
Supplement: sj-pdf-4-mde-10.1177_23821205211000356 – Supplemental material for A Systematic Scoping Review on Portfolios of Medical Educators [file sj-pdf-4-mde-10.1177_23821205211000356.pdf]

Supplementary File 4. Summary of Key Findings

No.	Author	Year	Title	MERSQI	COREQ	Background	Theoretical Approach and Methods	Main Empirical Findings	Insights Drawn
1	Baldwin, C., Chandran, L. and Gusic, M.	2011	Guidelines for evaluating the educational performance of medical school faculty: priming a national conversation	-	-	The academic community needs a sound framework for the promotion and advancement of educators. TheGroup on Educational Affairs of the Association of American Medical Colleges organized a consensus conference that affirmed the use of five domains for documenting the quantity and quality of scholarly engagement in educational activities: teaching, curriculum, advising/mentoring, educational leadership/administration, and learner assessment.	NA	In this article, we offer detailed guidelines to evaluate these five domains of educator performance and the essential elements of scholarly activity. The guidelines are adapted from our developmental educator portfolio template and educator portfolio analysis tool, previously published inMedEdPORTAL. A short tool for educator performance evaluation that summarizes items in the guidelines is proposed for discussion.	We have proposed itemized guidelines for the evaluation of educator performance to encourage thoughtful and consistent decision making about the promotion of faculty members whose careers are focused primarily on education. These guidelines include 24 items, divided among the five standard domains of education (19 items) and the standard elements

									of scholarly activity in education that apply across all domains (5 items).
2	Bannard-Smith, J., Bishop, S., Gawne, S. and Halder, N.	2012	Twelve tips for junior doctors interested in a career in medical education	-	-	As with the many specialities within medicine, the expanding field of medical education is becoming a more formal and professionalised arena	NA	Twelve practical suggestions on how junior doctors can nurture their portfolios of evidence and subsequent future careers in medical education	The developing world of medical education is becoming evermore structured and professionalised. Educators of the future will need to demonstrate evidence of their knowledge, skills and experience in the field in order to effectively assume and perform educational roles.
3	Bhargava, P., Patel, V. B., Iyer, R. S., Moshiri, M., Robinson, T.	2015	Academic portfolio in the digital era: organizing and maintaining a	-	-	The academic portfolio has become an integral part of the promotions process. Creating and maintaining an academic	NA	In this article, we describe an alternative way to efficiently organize an academ-ic	To our knowledge, we are the first to propose the novel role of reference

	J., Lall, C. and Heller, M. T.		portfolio using reference managers			portfolio in paper-based or web-based formats can be a cumbersome and time-consuming task. In this article, we describe an alternative way to efficiently organize an academic portfolio using a reference manager software, and discuss some of the afforded advantages. The reference manager software Papers (Mekentosj, Amsterdam, The Netherlands) was used to create an academic portfolio. The article outlines the key steps in creating and maintaining a digital academic portfolio. Using reference manager software (Papers), we created an academic portfolio that allows the user to digitally organize clinical, teaching, and research accomplishments in an		portfolio using a reference manager software, and discuss some of the afforded advantages. The reference manager software Papers (Mekentosj, Amsterdam, The Netherlands) was used to create an academic portfolio. The article outlines the key steps in creating and maintaining a digital academic portfolio. Using reference manager software (Papers), we created an academic portfolio that allows the user to digitally organize clinical, teaching, and research accomplishments in an indexed library enabling efficient updating, rapid retrieval, and easy	manager software, particularly Papers, in the creation of an academic portfolio. This method of digital portfolio creation is applicable not only in radiology but also across all medical specialties. As the digital era continues to permeate medicine, we anticipate greater reliance on such digital platforms in the future.

						indexed library enabling efficient updating, rapid retrieval, and easy sharing. To our knowledge, this is the first digital portfolio of its kind.		sharing. To our knowledge, this is the first digital portfolio of its kind.	
4	Blake, K.	2001	The daily grind--use of log books and portfolios for documenting undergraduate activities	-	-	In recent years, teaching portfolios have been developed as a way for faculty to document teaching scholarship, accomplishments, skills, and strategies. Teaching portfolios provide a means for teachers to demonstrate their teaching achievements and display their best work. I believe that many of us would have bene®ted from the experience of keeping a learning portfolio in our undergraduate and postgraduate years, both as a means of developing the habit of maintaining one and also as a means	NA		

						of examining our experiences and using the information as a basis for ongoing learning and improvement. Perhaps we should all undertake logging or portfolio keeping ourselves			
5	Chandran, L., Gusic, M., Baldwin, C., Turner, T., Zenni, E., Lane, J. L., Balmer, D., Bar-On, M., Rauch, D. A., Indyk, D. and Gruppen, L. D.	2009	Evaluating the performance of medical educators: a novel analysis tool to demonstrate the quality and impact of educational activities	-	11	Traditional promotion standards rely heavily on quantification of research grants and publications in the curriculum vitae. The promotion and retention of educators is challenged by the lack of accepted standards to evaluate the depth, breadth, quality, and impact of educational activities. The authors sought to develop a practical analysis tool for the evaluation of educator portfolios (EPs), based on measurable outcomes that allow reproducible analysis of the quality and impact of educational activities.	The authors, 10 veteran educators and an external expert evaluator, used a scholarly, iterative consensus-building process to develop the tool and test it using real EPs from educational scholars who followed an EP template. They revised the template in parallel with the analysis tool to ensure that EP data enabled valid and reliable evaluation. The authors created the EP template and analysis tool for	The analysis tool combines 18 quantitative and 25 qualitative items, with specifications, for objective evaluation of educational activities and scholarship.	The authors offer this comprehensive, yet practical tool as a method to enhance opportunities for faculty promotions and advancement, based on well-defined and documented educational outcome measures. It is relevant for clinical educators across disciplines and across institutions. Future studies

							scholar and program evaluation in the Educational Scholars Program, a three-year national certification program of the Academic Pediatric Association.		will test the interrater reliability of the tool, using data from EPs written using the revised template.
6	Chandran, L., Gusic, M. E., Lane, J. L. and Baldwin, C. D.	2017	Designing a National Longitudinal Faculty Development Curriculum Focused on Educational Scholarship: Process, Outcomes, and Lessons Learned	6.5	9	Clinical educators at U.S. academic health centers are frequently disadvantaged in the academic promotion system, lacking needed faculty development, mentoring, and networking support. Intervention: In 2006, we implemented the national Educational Scholars Program to offer faculty development in educational scholarship for early career educators in pediatrics. We aimed to provide them with skills, experience, and initial success in educational scholarship	In 2006, we implemented the national Educational Scholars Program to offer faculty development in educational scholarship for early career educators in pediatrics. We aimed to provide them with skills, experience, and initial success in educational scholarship and dissemination. The 3-year curriculum is delivered in interactive sessions at the annual pediatric academic	In 10 years, the Educational Scholars Program has enrolled 172 scholars in 8 cohorts; 94 have graduated so far. We describe how formative evaluation guided curriculum refinement and process improvement. Summative evaluations show that faculty and scholars were satisfied with the program. Participant outcomes from Cohort 1, assessed at Kirkpatrick's four	Curriculum building is a dynamic process of ongoing evaluation and modification. Our program benefited from designing an integrated and focused curriculum, developing educational principles to guide program improvements, creating curricular tools to help learners organize and

						and dissemination. The 3-year curriculum is delivered in interactive sessions at the annual pediatric academic meetings and online intersession modules. Curriculum content progresses from educational scholarship and implementing scholarly projects to dissemination and professional networking. Intersession modules address project planning, building an educator portfolio, reviewing the literature, using technology, authorship, and peer review. Concurrently, all scholars must complete a mentored educational project and demonstrate national dissemination of a peerreviewed product to obtain a Certificate of Excellence in Educational Scholarship.	meetings and online intersession modules. Curriculum content progresses from educational scholarship and implementing scholarly projects to dissemination and professional networking. Intersession modules address project planning, building an educator portfolio, reviewing the literature, using technology, authorship, and peer review. Concurrently, all scholars must complete a mentored educational project and demonstrate national dissemination of a peer- reviewed product to obtain a Certificate of	levels of evaluation, demonstrate increases in scholarly productivity, leadership activities, and academic promotions.	document their efforts, supporting project-based learning with expert mentoring, and facilitating peer and faculty networking and collaboration. A national, longitudinal faculty development program can support growth in academic knowledge and skills, promote professional networking, and thereby enrich educators' career opportunities.

							Excellence in Educational Scholarship. Context: The setting of this study was a national, longitudinal, cohort-based faculty development program built within the Academic Pediatric Association, a 2,000-member professional organization.		
7	Dalton, C. L., Wilson, A. and Agius, S.	2018	Twelve tips on how to compile a medical educator's portfolio	-	-	Medical education is an expanding area of specialist interest for medical professionals. Whilst most doctors will be familiar with the compilation of clinical portfolios for scrutiny of their clinical practice and provision of public accountability, teaching portfolios used specifically to gather and demonstrate medical education activity remain	NA	A medical educator's portfolio is an essential resource for those interested in pursuing a career in medical education. A portfolio may take many forms, adopt a variety of structures and be put to a number of uses. In all cases it should be a dynamic document that promotes self-assessment and	Through this article, we have sought to demonstrate that compiling a medical educator's portfolio does not need to be onerous. If used regularly, it can become the most helpful of colleagues: one who remembers

						uncommon in many non-academic settings. For aspiring and early career medical educators in particular, their value should not be underestimated. Such a medical educator's portfolio (MEP) is a unique compendium of evidence that is invaluable for appraisal, revalidation, and promotion. It can stimulate and provide direction for professional development, and is a rich source for personal reflection and learning. We recommend that all new and aspiring medical educators prepare an MEP, and suggest twelve tips on how to skillfully compile one.		reflection, and allows users to plan their professional development.	all your achievements and successes whilst supporting you in over- coming challenges that may arise in your career.
8	Dolmans, D. H. and Tigelaar, D.	2012	Building bridges between theory and practice in medical education using a design-	-	-	Medical education research has grown enormously over the past 20 years, but it does not	NA		

			based research approach: AMEE Guide No. 60			sufficiently make use of theories,<br>according to influential leaders and researchers in this field. In this AMEE Guide, it is argued that design-based research (DBR) studies should be conducted much more in medical education design research because these studies both advance the testing and refinement of theories and advance educational practice. In this Guide, the essential characteristics of DBR as well as how DBR differs from other approach such as formative evaluation are explained. It is also explained what the pitfalls and challenges of DBR are. The main challenges deal with how to insure that DBR studies reveal findings that are of a broader relevance than the local situation and how to			

						insure that DBR contributes toward theory testing and refinement. An example of a series of DBR studies on the design of a teaching portfolio in higher education that is aimed at stimulating a teacher's professional development is described, to illustrate how DBR studies actually work in practice. Finally, it is argued that DBR-studies could play an important role in the advancement of theory and practice in the two broad domains of designing or redesigning work-based learning environments and assessment programs.			
9	Goliath, Cheryl Lynn	2010	Diffusion of an e-portfolio to assist in the self-directed learning of physicians: An exploratory study	-	-	Professional societies in the field of medicine have recommended that the<br><br>traditional model for lifelong medical learning,	A single case study comprised of nine physicians was used to examine the factors involved when the diffusion of an e-portfolio was	The results were reviewed and key points were identified: (1) increased scores on the SDLRS from the pre-test to the post-	This study examined the impact of implementing an e-portfolio system within an Internal Medicine

						which had previously focused on attendance at weeklong didactic continuing medical education (CME) courses, should be replaced by individualized study. Self-directed and practice-linked learning are well accepted in principle, but techniques that enhance their execution have not been well described as a means of obtaining CME. While there is considerable innovation underway in undergraduate, postgraduate and continuing medical education; the ultimate goal of these initiatives is to develop an electronic infrastructure that would gather experiences, outcomes and evidence of competencies appropriate to the education, practice level and daily activities	introduced. The participants were studied using interviews, the Stages of Concern (SoCQ) and the Self-Directed Learning Readiness Scale (SDLRS) questionnaires, four-week trial period of I-Webfolio and field notes based upon observations during the study.<br><br>In the first step, knowledge; an informational meeting with the participants provided the necessary information to them to allow for a basic knowledge of the innovation and what the study would entail. There was an opportunity for questions and each	test would indicate that the diffusion of I-Webfolio was effective in improving the self-directed learning of the participants; (2) factors other than Board certification, age, teaching faculty, association with a group practice, academic appointment and size of institution should be considered when introducing a new innovation to physicians; (3) merely possessing the self-directed learning ability is not sufficient to maintain engagement of the innovation; (4) the efficacy of I-Webfolio proved to be an effective educational strategy based upon the	physician practice as a means of developing a process of life-long learning and professional development for the participants. While no clear-cut user profile was identified, that fact serves as reinforcement that no two adult learners are alike in their abilities, perception and learning styles. As the introduction of I-Webfolio moves on to a new group of learners, the information learned from this study will dramatically impact the diffusion process

						of individual physicians; compile such data in relation to learning expectations and performance of other comparable learners; and present it to the individual to stimulate and guide self-assessment and self-improvement.	of the participants was encouraged to approach the researcher with any questions that might arise throughout the study. The close working proximity of the researcher to the participants provided opportunity for adequate communication. In the next step, persuasion; the informational meeting also provided the participants with the findings from the review of the literature. The consensus was that an alternative method for supporting physician CME needed to be investigated and that e-portfolios had been successfully	increased SDLRS scores as well as the SoCQ scores following a predictable pattern of users who have engaged a new innovation; (5) factors influencing the adoption of technology for the purpose of self-directed learning included time limitations from reduced work schedules or increased work demands, security issues and long-term access.<br><br>The results were analyzed to identify patterns or themes that appeared. One such pattern emerged, in that; all entries in the e-portfolio were almost	used in the future.

							implemented in other institutions. In step three, decision; all nine physicians volunteered to participate in the study and all remained engaged in the study through conclusion. In step four, implementation; I-Webfolio was used by the participants for a four-week trial period. Finally, in step five, confirmation; at the conclusion of the study, eight of the nine physicians indicated they would continue using I-Webfolio and one physician stated they would not .	exclusively in the Medical Knowledge component. A theme that became apparent was that none of the physicians in the study have developed a self- directed learning plan. The results of this study suggested that adoption of technology is multifactoral and there was no consistent individual profile that predicted optimal adoption of the innovation; however, increased scores on the SDLRS would indicate that the diffusion of I-Webfolio was effective in improving the self-directed learning of the participants.	

10	Ingrassia, A.	2013	Portfolio-based learning in medical education	-	-	The use of portfolios has rapidly expanded in recent years and is now relevant to a number of different aspects of professional practice in medicine in general and psychiatry in particular, including training, appraisal and revalidation as well as continuing professional development. In this article I will examine the background of important changes and new trends in medical education on which the increasing use of portfolios is based, their potential value as learning and assessment tools, and some of the challenges and dilemmas associated with their use.	NA	-	The use of portfolios as an example of 'authentic', real-life assessments gives us yet another opportunity to do just that. In addition, the use of portfolios calls for medical educators to re-think some of the paradigms on which traditional examinations and appraisals have been based, to make room for a different set of educational values and prioritise learning from reflective practice which 'accepts the subjectivity of data and interpretations,

									and focuses on individual insights and developments [...] values creativity and, importantly, allows and understands the possibility of being wrong' (Pitts 2010: p. 104).
11	Izatt, Susan	2007	Educational Perspectives: Portfolios: The Next Assessment Tool in Medical Education?	-	-	Medical education is undergoing significant change at all levels. Selfdirected learning and reflection are becoming essential ingredients of both graduate medical training and postgraduate professional development. Recognition is growing for the need to incorporate the concepts of andragogy into physician education, concepts emphasizing that adult learners are	NA	-	Quality health care relies on medical professionals who have a solid educa- tional cornerstone that is maintained through lifelong learning and reflec- tion. The public demands compe- tency of those who provide health care. The assessment of

						self-directed and take responsibility for their own educational choices. The demonstration of competency for assessment ofthe individual and the educational program has received increased attention in the Accreditation Council for Graduate Medical Education (ACGME) Outcome Project. (1) Assessment and documentation of self-directed learning, reflection, and competency are more difficult than traditional methods that have included standardized testing and conference attendance. The portfolio has been advocated as a tool for collecting and presenting these elements oflearning at all levels of medical training.			such charac- teristics through all stages of medical training and practice is essential but difficult. The portfolio offers one po- tential tool to help with this process.

12	Lawson, M., Nestel, D. and Jolly, B.	2004	An e-portfolio in health professional education	-	2	This report describes an e-portfolio developed to support a graduate certificate in health professional education. The course addresses the skills and knowledge of teachers working in health and encourages the development of a professional approach to teaching practice. The first multiprofessional cohort completed the course in 2003. The e-portfolio was developed primarily to enable the preparation, sharing and submission of assessment items in the actual workplace of the course participants and to facilitate peer and tutor feedback.	Interviews and Written Evaluations	Interviews and written evaluations were used to evaluate thee-portfolio from participant and tutor perspectives. Participants started the programme with diverse levels of confidence, experience and competence inworking with web-based resources. We overestimated their capacity in this respect and had difficulties in preparing them to use the e-portfolio. Participants have since reported that the e-portfolio was relatively easy to use. They have expressed mixed levels of confidence in the technology. Writing up reflections was an unfamiliar concept to	

								these participants and his created additional challenges. Few participants reported using the personal journal space, either because they were unfamiliar with the concept or because computers were not always available in work settings. Other difficulties related to technical aspects of thee-portfolio. For the second course unit a parallel e-portfolio development site was operated to enable identification, replication and management of technical difficulties. This proved valuable for articulating specific problems and solutions. Tutors appreciated the easy electronic access to	

								all student assessments, the reduction in paper and the central repository of course information. They also valued the introduction of a new educational method with a transparent and participatory evaluation methodology	
13	Sidhu, N. S.	2015	The teaching portfolio as a professional development tool for anaesthetists	-	-	A teaching portfolio (TP) is a document containing a factual description of a teacher's teaching strengths and accomplishments, allowing clinicians to display them for examination by others. The primary aim of a TP is to improve quality of teaching by providing a structure for self-reflection, which in turn aids professional development in medical education. Contents	NA	The primary aim of a TP is to improve quality of teaching by providing a structure for self-reflection, which in turn aids professional development in medical education. Contents typically include a personal statement on teaching, an overview of teaching accomplishments and activities, feedback from colleagues and	This article is intended to facilitate the preparation of a TP by clinicians whose primary role may not be an academic one. As Weeks writes, "The portfolio is a comprehensive collection of data and, like a good wine cellar, the longer it is kept and developed, the more

						typically include a personal statement on teaching, an overview of teaching accomplishments and activities, feedback from colleagues and learners, a reflective component and some examples of teaching material. Electronic portfolios are more portable and flexible compared to paper portfolios. Clinicians gain the most benefit from a TP when it is used as a tool for self-reflection of their teaching practice and not merely as a list of activities and achievements. This article explains why and how anaesthetists might use a TP as a tool for professional development in medical education.		learners, a reflective component and some examples of teaching material. Electronic portfolios are more portable and flexible compared to paper portfolios. Clinicians gain the most benefit from a TP when it is used as a tool for self-reflection of their teaching practice and not merely as a list of activities and achievements. This article explains why and how anaesthetists might use a TP as a tool for professional development in medical education.	effective it will be as a tool for professional development"5. Improvement in teaching skills should be seen as the main benefit, in keeping with the overall aim to promote excellence and scholarship in medical education within anaesthesia as a specialty. TPs are clearly not for everyone and the evidence for their use is not wholly conclusive. No research has been done specifically looking at improvement in teaching among anaesthetists and this would be difficult to

									measure. Nonetheless, the utility of reflective learning and a log of teaching activities with feedback may justify its use among anaesthetists who consider teaching an integral component of their duties. The use of a TP by a clinician-educator, although to be encouraged and facilitated, should remain voluntary. Mandatory implementation among anaesthetists would likely result in superficial use in order to fulfil set

									requirements. In order for them to gain maximum benefit, anaesthetists should themselves see the utility of maintaining a TP and use it for self-reflection of their teaching practice. To forgo the reflective component would render the portfolio as simply a list of activities and achievements.
14	Simpson, D., Hafler, J., Brown, D. and Wilkerson, L.	2004	Documentation systems for educators seeking academic promotion in U.S. medical schools	10	16	To explore the state and use of teaching portfolios in promotion and tenure in U.S. medical schools.	A two-phase qualitative study using a Web-based search procedure and telephone interviews was conducted. The first phase assessed the penetration of teaching portfolio-like systems in U.S.	The Phase 1 search of U.S. medical schools' Web sites revealed that 76 medical schools have Web-based access to information on documenting educational activities for promotion. A	The number of medical schools whose promotion packets include portfolio-like documentation associated with a faculty member's excellence in educa-tion has

							medical schools using a keyword search of medical school Web sites. The second phase examined the current use of teaching portfo-lios in 16 U.S. medical schools that reported their use in a survey in 1992. The individual designated as having primary responsibility for faculty appointments/promo-tions was contacted to participate in a 30 – 60 minute interview.	total of 16 of 17 medical schools responded to Phase 2. All 16 continued to use a portfolio-like system in 2003. Two documentation cate-gories, honors/awards and philosophy/personal statement regarding education, were included by six more of these schools than used these categories in 1992. Dissemination of work to colleagues is now a key inclusion at 15 of the Phase 2 schools. The most common type of evidence used to document education was learner and/or peer ratings with infrequent use of outcome measures and internal/external review.	increased by more than 400% in just over ten years. Among early-responder schools the types of docu-mentation categories have increased, but students' ratings of teaching remain the primary evidence used to docu-ment the quality or outcomes of the educational efforts reported.

15	Swanwick, T., McKimm, J. and Clarke, R.	2010	Introducing a professional development framework for postgraduate medical supervisors in secondary care: considerations, constraints and challenges	-	11	As the duration of postgraduate training becomes shorter, direct patient contact time is reduced, and supervision becomes more distributed, there is a move to 'professionalise' postgraduate medical education. This paper reports an initiative in one postgraduate training institution (the London Deanery) to develop and introduce a developmental framework and system of portfolio-based review of educational supervisors in the secondary care setting.	16 acute, mental health, foundation and primary-care Trusts participated in a pilot project, which was subsequently evaluated using focus groups and a semistructured questionnaire.	Thematic analysis of transcripts identified a number of considerations, constraints and challenges, important observations given the current policy intention of the UK health departments to introduce mandatory training and performance review for educational supervisors.	This pilot study shows that such a process can be implemented at local level if facilitated by a clear and unambiguous developmental framework that can be applied flexibly across all specialities. Systems of review also need to be simple and straightforward, take into account existing appraisal processes, and simultaneously address issues of motivation, recognition and reward.
16	Taylor, B. B., Parekh, V., Estrada, C. A.,	2013	Documenting Quality Improvement and Patient Safety Efforts: The	-	-	Physicians increasingly investigate, work, and teach to improve the quality of care and safety of care delivery. The	The Society of General Internal Medicine Academic Hospitalist Task Force sought to	The portfolio has eight categories including: (1) a faculty narrative, (2) leadership and	The authors offer this comprehensive, yet practical tool as a method to

	Schleyer, A. and Sharpe, B.		Quality Portfolio. A Statement from the Academic Hospitalist Taskforce			Society of General Internal Medicine Academic Hospitalist Task Force sought to develop a practical tool, the quality portfolio, to systematically document quality and safety achievements. The quality portfolio was vetted with internal and external stakeholders including national leaders in academic medicine. The portfolio was refined for implementation to include an outlined framework, detailed instructions for use and an example to guide users. The portfolio has eight categories including: (1) a faculty narrative, (2) leadership and administrative activities, (3) project activities, (4) education and curricula, (5) research and scholarship, (6) honors, awards, and	develop a practical tool, the quality portfolio, to systematically document quality and safety achievements. The quality portfolio was vetted with internal and exter-nal stakeholders including national leaders in aca-demic medicine. The portfolio was refined for implementation to include an outlined framework, detailed instructions for use and an example to guide users.	administrative activities, (3) project activities, (4) education and curricula, (5) research and scholar-ship, (6) honors, awards, and recognition, (7) train-ing and certification, and (8) an appendix.	document quality and safety activ-ities. It is relevant for physicians across disciplines and institutions and may be useful as a standalone document or as an adjunct to traditional promotion documents. As the Next Accreditation System is implemented, academic medical centers will require faculty who can teach and implement the systems-based practice requirements. The quality portfolio is a method to document quality

						recognition, (7) training and certification, and (8) an appendix. The authors offer this comprehensive, yet practical tool as a method to document quality and safety activities. It is relevant for physicians across disciplines and institutions and may be useful as a standalone document or as an adjunct to traditional promotion documents. As the Next Accreditation System is implemented, academic medical centers will require faculty who can teach and implement the systemsbased practice requirements. The quality portfolio is a method to document quality improvement and safety activities.			improvement and safety activities.
17	Tigelaar, D. E., Dolmans, D. H., de Grave, W. S.,	2006	Portfolio as a tool to stimulate	-	12	Portfolios are increasingly being used to stimulate teachers' reflections. Frameworks	Firstly, the teachers were asked to describe the course of their teaching	Outcomes of written portfolio assignments were collected and analysed to identify	his study might help teachers and their trainers and coaches to

	Wolfhagen, I. H. and van der Vleuten, C. P.		teachers' reflections			for reflection on teaching often emphasize competencies and behaviours. However, other aspects of teacher functioning are also important, such as the teaching environment and individual teachers' beliefs, professional identity and mission. In a study among five medical school teachers, we explored how a portfolio stimulated reflections on the various aspects of teaching functioning. Outcomes of written portfolio assignments were collected and analysed to identify examples of reflections on the various aspects of teacher functioning. Examples of reflections on all aspects of teacher functioning were found, although examples of reflections on competencies were easier	career. They were also asked to describe critical incidents in their development as a teacher, set learning goals, select artefacts in evidence of their teaching activities, compose a profile of a good teacher and reflect on that. The final assign-ment required them to compose a reflective portfolio, using the previous assignments as points of departure. The assignments were intended to stimulate reflection on all aspects of teacher functioning as defined in Korthagen's model. However, the critical incidents assignment was focused on the environment,	examples of reflections on the various aspects of teacher functioning. Examples of reflections on all aspects of teacher functioning were found, although examples of reflections on competencies were easier to find than those on beliefs, identity and mission.	recognize different aspects of teacher functioning when discussing portfolios for professional development purposes. However, further development of assignments and other methods to stimulate reflections on beliefs, identity and mission are needed. Furthermore, apart from the content of teachers' reflections, teachers' reflection processes should be researched, as well as the effects of portfolio meetings with

						to find than those on beliefs, identity and mission. This study might help teachers and their trainers and coaches to recognize different aspects of teacher functioning when discussing portfolios for professional development purposes. However, further development of assignments and other methods to stimulate reflections on beliefs, identity and mission are needed. Furthermore, apart from the content of teachers' reflections, teachers' reflection processes should be researched, as well as the effects of portfolio meetings with peers and coaches.	behaviour, and competencies. Table 1 presents brief descriptions of the assignments.		peers and coaches.
18	Tigelaar, D. E., Dolmans, D. H., de Grave, W. S., Wolfhagen, I.	2006	Participants' opinions on the usefulness of a teaching portfolio	-	12	Whether teaching portfolios achieve their aim of stimulating teachers' professional development is	Five teachers and their personal coaches were individually interviewed about	The teaching roles were helpful in analy-sing teaching, but the assignments and the conver-sation	The portfolio structure and social interactions, especially

	H. and van der Vleuten, C. P.					favourably affected by the incorporation ofa balanced structure and effective social interactions, such as coaching. We explored teachers' experiences with a teaching portfolio that was structured by teaching roles, portfolio assignments and conversation protocols. The related social interactions consisted of meetings with peers and personal coaches. Teachers' and coaches' opinions were sought about the benefits and the enabling and disabling factors ofthe portfolio structure and the social interactions involved.	their experiences with the portfolio. Protocols were analysed using a top-down strategy.	protocols were too detailed and directive. The social interactions were perceived as very valuable for professional development, particularly the meetings with personal coaches.	personal coaching, appeared to be helpful for teachers in analysing their teaching practice. The assignments and protocols, however, were too detailed and directive. This may be resolved by using assignments and conversation protocols more flexibly and enabling adjustment to personal learning needs. The high appreciation of personal coaching might be related to the freedom for teachers to choose their own coach. The

									results can be used by portfolio designers as guidelines for how to achieve a balanced structure and effective social interactions and how these might be combined to increase the benefits of working with a portfolio to teachers.
19	Zobairi, S. E., Nieman, L. Z., Cheng, L.	2008	Knowledge and Use of Academic Portfolios Among Primary Care Departments in U.S. Medical Schools	9	-	Portfolios are used to summarize and communicate the academic accomplishments of faculty seeking career development and promotion.	we assessed knowledge and use of the portfolio in a cross sectional survey of 435 aca-demic chairpersons of family medicine and division directors of general internal medicine and general pediatrics.	Of the 241 respondents (55% response rate), 69% reported knowledge of portfolios and 55% used portfolios. Satisfaction with the source of information on a faculty candidate was greater when portfolios were used (85.5% of respondents) than when they were not	Results suggest that leaders of primary care departments use portfolios to pro-mote the career development of their faculty and that portfolio use is influenced by attitudes and training.

								used (67.3% of respondents; p = .01). Increased knowledge and use of portfolios was associated with respondents working at institutions with academic fellowships/advanced education programs or who had fellowship training (ps = .01).	
20	Robert T Brodell, Murad Alam, David R Bickers	2003	The dermatologist's academic portfolio: a template for documenting scholarship and service	-	-	The clinician-teacher of dermatology often seeks recognition for academic efforts, the most visible of which is a university appointment with the potential for promotion. Success in achieving this goal requires careful planning to ensure effective involvement in academic pursuits that serve the mission of the university, and there must be concomitant documentation of this involvement. The	-	-	In summary, a well organized academic portfolio can make the difference in assuring a successful promotion process in the university setting. Such a portfolio with detailed information as described in this article is superior to a simple

						template in this article provides one format for organizing an academic portfolio that stimulates prospective documentation and enhances the possibility of academic advancement.			curriculum vitae in the promotion process.[11] An academic portfolio provides impor- tant information that is invaluable to committees that are responsi- ble for evaluating an individual's suitability for promotion or tenure, with the information needed to permit a fair and objective assessment of academic performance. If a university committee has its own format for a promotion package the portfolio template described here

									can readily be adapted to it.
21	Saee Deshpande, Suresh Chari, Usha Radke, Tapasya Karemore	2019	Evaluation of the educator's portfolio as a tool for self-reflection: Faculty perceptions	-	22	Preparing a teaching portfolio to document educational expertise has shown to be useful for both promotion and to stimulate faculty development. This article describes a study of the effectiveness of the Educator's Portfolio (EP) as a stimulus for faculty reflection about educational practice.	A sensitizing session of thirty faculty from medical, dental, and physiotherapy colleges on the same campus was conducted; faculty members were asked to complete and submit their EPs. Out of 30 members, 25 responded (83%). Semi-structured interviews of 25 faculties who prepared EPs were conducted, and a qualitative content analysis of the resulting protocols was completed to determine how the EP development process had promoted their reflection on education.	All the 25 faculty members indicated that reflection about education had occurred. Four categories of reflection emerged, namely, (a) lack of understanding regarding how to categorize their work; (b) lack of evidence of the effectiveness of educational activities; (c) reformulating educational practice; and (d) source of motivation and self-regulation.	The findings indicate that EP preparation serves as a tool for reflection on educational practice, which promotes faculty development.

22	David J Klocko	2010	The use of course portfolios to document the scholarship of teaching	-	-	One strategy that novice and experienced PA faculty can use to address the needs of the scholarship of teaching — particularly the institution's need to address faculty evaluation, tenure, and promotion — is to use a course portfolio. A course portfolio can be used in many other ways that are beneficial to the instructor, fellow faculty, and the PA program	-	-	Many universities across the country are integrating scholarship of teaching and excellence of teaching institutionally and across many academic disciplines. They report that course portfolios have contributed significantly in departmental accreditation, peer review during promotion, faculty development, and "develop[ment of] a vocabulary about how to discuss the intellectual work of the scholarship of teaching."

									They also report improved grant funding by faculty grant writers who have produced course portfolios as part of their scholarly work.6 In a similar fashion, the integration of course portfolios in PA education can help to advance our developing academy of PA educators and serve as a forum to share information, receive critique from colleagues, and generate questions and debate about our teaching.
23	Gloria J Kuhn	2004	Faculty development: The educator's	-	-	The educator's portfolio is of value for both university and	-	-	The educator's portfolio has

			portfolio: Its preparation, uses and value in academic medicine			community-based academic faculty. It can be used to document scholarly activity and teaching and to prepare for periodic evaluations. Many faculty members use it to assist them in managing their careers and to reflect on activities and teaching efforts to ensure continued growth in competency as teachers. Promotion and tenure committees of many institutions now use this document to aid them in making promotion decisions.			become a valuable tool for documenting the activities and accomplishments of educators. It can be used to assist in evaluation, setting of goals, and reflection and growth in faculty. Many institutions now use the portfolio to assist them in determining promotion decisions. Although no one is promoted simply because he or she creates

									310 Kuhn d THE EDUCATOR'S PORTFOLIO<br><br>a portfolio, the portfolio is an ideal vehicle to display<br><br>the quality and quantity of the educator's work in<br><br>a format that makes it easily accessible to evaluators<br><br>whether they are on a P/T committee or chair of<br><br>a department.
24	Neela Lamki, Mark Marchand	2006	The medical educator teaching portfolio: its compilation and potential utility	-	-	A revolution in thinking and redefinition of traditional scholarship by prioritising teaching skills and achievements	-	-	The portfolio described is meant to be a complete account of a teacher's thoughts, activities, and

						has led to the birth of the Teaching Portfolio, designed to carry a comprehensive and dynamic record of the teaching activities of the faculty. A teaching portfolio documents the faculty's teaching scholarship and effectiveness. It is a record of selected information on one's teaching achievements, skills and strategies and dynamically represents the faculty's growth, progress and teaching record. The author suggests a template for the Teaching Portfolio of a Medical Educator, which consists of four parts: (□) evaluation, (2) personal professional development, (3) learning			successes —a dynamic collection that is never finished, but rather actively supplemented. In its fullest capacity, a METP can be used for evaluation, reflection, continued learning and, of paramount importance, improved teaching. Compiled with the aid of a mentor and with a well-referenced appendix, the METP becomes a more objective measure of an

						processes and (4) an appendix.			educator’s value. Such an assessment tool is not meant to be used alone, but rather in conjunction with other established methods including the standard CV and direct observation where possible. As portfolios become standardized, their value and ease of use increases. Each Institution must create its own template that the faculty can use

									in the creation of his/her METP. We offer in this article<br><br>an example that can be used as a step toward developing a standardized template for medical institutions.<br><br>Potentially it could be a stimulant or a future model for<br><br>an improved universally acceptable medical educator's<br><br>teaching portfolio.
25	Kadriye O Lewis, Raymond C Baker	2007	The development of an electronic educational portfolio: an	-	-	A growing body of literature shows that many universities and colleges are making	Guidelines for the development of an educational portfolio focusing on medical	-	We believe that portfolio development is a valuable

			outline for medical education professionals			educational portfolios part of their faculty assessment and student's learning plan for both undergraduate and graduate programs. We provide an outline for the development of a medical educator's portfolio, including an electronic version.	education are provided, including design, format, and content. An electronic version of the portfolio, which combines flexibility and ease of revision, is also described, including formats for publication and distribution. Student reflections on the e-portfolio are presented, and potential applications of the e-portfolio in medical education are described.		application that provides rich documentation of participants' educational history, accomplishments, and intellectual property as related to their professional learning and growth.
26	Kim Little-Wienert, Mark Mazziotti	2017	Twelve tips for creating an academic teaching portfolio	-	-	An academic teaching portfolio is not only a requirement at many academic teaching institutions, but it is also important in a medical educator's growth and development through documentation, reflection, evaluation, and change. Creating an	-	-	An academic teaching portfolio allows for multiple sources of input to document educational expertise, while also supporting promotion based

						academic portfolio may appear daunting at first but with careful advanced preparation, organized evidence collection of your educational work, proof of scholarship, and thorough documentation of self-reflection and change, you can produce a successful product that accurately represents your educational beliefs, accomplishments, and growth throughout your career. This article provides medical educators with twelve steps for creating a successful academic teaching portfolio.			on important educational endeavors. The twelve tips we have outlined here provide the framework to best document and present an individual's educational philosophy, goals, and accomplishments. The academic teaching portfolio is an ideal presentation of your accomplishments to support promotion, and it also allows for professional development

									through self-directed learning and reflection. It is, thus, a vital tool for the medical educator.
27	Andrew M Luks, Michi Yukawa, Helen Emery	2009	Disseminating best practices for the educator's portfolio	6	-	The clinician-educator pathway is increasingly recognised as a distinct career track at many institutions. Despite its growing popularity and previous efforts to delineate the means by which clinician-educators should document their excellence and scholarship, the pathway remains poorly defined at many institutions; promotions criteria are not specified as well as they are for doctor-scientists, and many clinician-educators lack information about how to	We conducted an online survey of 294 clinician-educators and a paper-based survey of 17 department chairs to define current portfolio practices at our institution. After a literature review and an informal survey of practices at other institutions, we created a template for the educator’s portfolio and an assessment tool for portfolio evaluation. We presented our work to the dean of the School of Medicine and the	Ten of 17 clinical departments responded to the survey; only two of these departments had formal portfolio development guidelines in place and none had formal evaluation criteria. A total of 125 faculty members participated in the survey (response rate 43%); 48% of these respondents did not know the difference between an educator’s portfolio and a promotions packet, and 86% had not received any	-

						document the nature and quality of their work.	chairpersons of the clinical departments at our institution. We conducted a lecture at a workshop for new faculty members and presented a separate half-day faculty development workshop on the educator's portfolio, during which we disseminated the template, provided guidance regarding the development and evaluation process, and provided education on key aspects of effective portfolios, including writing an effective teaching philosophy statement and demonstrating educational scholarship.	formal information from their departments regarding portfolio development or the evaluation process. Fifty-five people attended the half-day faculty development workshop, the largest attendance ever recorded at a faculty development workshop at our institution. All attendees received a binder with the portfolio template and detailed instructions about the information to be included in each section and the best means for presenting that information. Attendees completed evaluations of the workshop and gave it an overall score of 4.7 (0 = lowest, 5	

								= highest). The workshop also received high scores for clarity of content (4.67), organisation (4.67) and relevance of content to clinician-educators (4.73). A formal committee evaluating the promotions process at our institution has incorporated our template into its specific recommendations to the dean of the School of Medicine at our institution.	
28	Anh-Vu Ngo, Mahesh M. Thapa	2018	Documenting your career as an educator electronically	-	-	Academic radiologists on the clinician–educator track are required to provide a teaching portfolio as well as a curriculum vitae (CV) for academic promotion. The specific components of a teaching portfolio vary slightly depending on the	-	-	Online electronic portfolios are a great tool to showcase one-self for academic promotion and allow for multimedia formats that can be easily updated

						institution. Traditionally academicians have collected the necessary documentation in paper format, often stuffing them in a binder or a desk drawer. However in the digital age most academic works are readily available in electronic format and can be accessed online. An electronic portfolio is the ideal method to efficiently organize this digital material.			and accessed. Depending on the needs of an institution or individual, electronic portfolios can be standardized or infinitely customized. With many resources available to create an electronic online portfolio, we suggest using a free service initially and avoiding wiki-type sites. This allows users to discover their individual needs and preferences before committing to paid upgrades or services that might not be necessary. The

									authors' personal recommendation for a beginner is Google Sites.
29	Michael S Ryan, Constance Tucker, Deborah DiazGranados, Latha Chandran	2019	How are clinician-educators evaluated for educational excellence? A survey of promotion and tenure committee members in the United States	10	-	In recent years, educational leaders have proposed domains of educational excellence and corresponding metrics to objectively measure contributions of clinician-educators for promotion and tenure (P&T). The purpose of this study was to explore whether P&T committees in United States (US) have incorporated these recommendations into practice.	The authors conducted a survey of P&T leaders across institutions in US. Items included questions related to institutional tracks for P&T, domains included in promotional packets, metrics for their measurement, and use of an Educator's Portfolio (EP).	Respondents from 55 institutions completed the survey. The presence of a teaching academy/society was associated with the presence of a promotion track for clinician-educators ($p = 0.04$). Only teaching activities (91%), assessment of learners (55%), and educational scholarship (51%) were required by a majority of institutions. Few institutions used objective methods for measuring impact and less than half (47%) required an EP.	These results highlight both progress in the recognition of clinician-educators while also suggesting discordance in the perspective of educational leaders and the practice of P&T committees. The authors advocate for establishing a national community of expert medical educators who may assist P&T committees in adopting consensus-based criteria and metrics to evaluate

									clinician-educators' contributions.
30	Kanade Shinkai, Chen Amy Chen, Brian S Schwartz, Helen Loeser, Cynthia Ashe, David M Irby	2018	Rethinking the Educator Portfolio: An Innovative Criteria-Based Model	-	-	Academic medical centers struggle to achieve parity in advancement and promotions between educators and discovery-oriented researchers in part because of narrow definitions of scholarship, lack of clear criteria for measuring excellence, and barriers to making educational contributions available for peer review. Despite recent progress in expanding scholarship definitions and identifying excellence criteria, these advances are not integrated into educator portfolio (EP) templates or curriculum vitae platforms. From 2013 to 2015, a working group from the Academy of Medical Educators (AME) at the University	-	The EP 2.0 template was integrated into the AME application, resulting in high overall satisfaction among candidates and the selection committee and positive feedback on the template's transparency, ease of use, and streamlined format. In 2016, the EP 2.0 template was integrated into the campus-wide curriculum vitae platform and academic advancement system. The authors plan to increase awareness of the EP 2.0 template by educating promotions committees and faculty at UCSF and	The authors plan to increase awareness of the EP 2.0 template by educating promotions committees and faculty at UCSF and partnering with other institutions to disseminate it for use. They also plan to study the impact of the template on supporting educators by making their important scholarly contributions available for peer review, providing guidance for professional development, and

						of California, San Francisco (UCSF) designed a streamlined, criteria-based EP (EP 2.0) template highlighting faculty members' recent activities in education and setting rigorous evaluation methods to enable educational scholarship to be objectively evaluated for academic advancement, AME membership, and professional development.		partnering with other institutions to disseminate it for use. They also plan to study the impact of the template on supporting educators by making their important scholarly contributions available for peer review, providing guidance for professional development, and decreasing disparities in promotions.	decreasing disparities in promotions.
31	Deborah Simpson, Ruth-Marie E Fincher, Janet P Hafler, David M Irby, Boyd F Richards, Gary C Rosenfeld, Thomas R Viggiano	2007	Advancing educators and education by defining the components and evidence associated with educational scholarship	-	11	This study aimed to establish documentation standards for medical education activities, beyond educational research, for academic promotion consistent with principles of excellence and scholarship.	In 2006 a Consensus Conference on Educational Scholarship was convened by the Association of American Medical Colleges (AAMC) Group on Education Affairs (GEA) to outline a set of documentation standards for use by	The 110 conference participants, including medical school deans, academic promotion committee members, department chairs, faculty and AAMC leaders, re-affirmed the 5 education activity categories (teaching, curriculum, advising	Educators' contributions to their institutions must be visible to be valued. The establishment of documentation standards for education activities provides the foundation for academic

							educators and academic promotion committees. Conference participants' work was informed by more than 15 years of literature on scholarship, educator portfolios and academic promotion standards.	and/or mentoring, education leadership and/or administration, and learner assessment), the contents of each category, and cross-category documentation standards. Educational excellence requires documentation of the quantity and quality of education activities. Documenting a scholarly approach requires demonstrating evidence of drawing from and building on the work of others, and documenting scholarship requires contributing work through public display, peer review and dissemination; both involve	recognition of educators.

								engagement with the community of educators. Implementation of these standards – quantity, quality and engagement with the education community – should occur in parallel with the development of an infrastructure to support educators, including sustained faculty development for educators, access to educational resources and journals, peer review mechanisms and consultation and support specific to each activity category.	
32	John V Thomas, Rupan Sanyal, Janis P O'Malley, Satinder P	2016	A guide to writing academic portfolios for radiologists	-	-	The academic educator's portfolio is a collection of materials that document academic performance and achievements, supplementing the	-	-	Although the CV remains the best executive summary of these

	Singh, Desiree E Morgan, Cheri L Canon					curriculum vitae, in order to showcase a faculty member's most significant accomplishments. A decade ago, a survey of medical schools revealed frustration in the nonuniform methods of measuring faculty's medical education productivity. A proposed solution was the use of an academic educator's portfolio. In the academic medical community, compiling an academic portfolio is always a challenge because teaching has never been confined to the traditional classroom setting and often involves active participation of the medical student, resident, or fellow in the ongoing care of the patient. Diagnostic radiology in addition requires a knowledge base that			types of activities, it cannot always convey the depth of a commitment or the importance of an activity or honor. In such cases,<br><br>a portfolio can provide the appropriate place to explain the contents of the CV. It should not be populated with repetitive<br><br>material from the CV but should selectively describe and expand<br><br>on the material. In the case of the radiology educator, the teaching portfolio can be an excellent vehicle

						encompasses basic sciences, imaging physics, technology, and traditional and molecular medicine. Teaching and performing research that involves this complex mix, while providing patient care that is often behind the scenes, provides unique challenges in the documentation of teaching, research, and clinical service for diagnostic radiology faculty. An academic portfolio is seen as a way to explain why relevant academic activities are significant to promotions committee members who may have backgrounds in unrelated academic areas and may not be familiar with a faculty member's work. The academic portfolio consists of teaching, research, and service portfolios. The			to convey the wide variety of teaching opportunities available to the radiologist that may not fit neatly in the CV. It can outline growth by showing the path from teaching at the local view box to developing curricula to teaching as an expert at regional, national, and international meetings. It is a place to provide evidence of datadriven changes in teaching style or

						teaching portfolio is a collection of materials that document teaching performance and documents the educator's transition to a more effective educator. A research portfolio showcases the most significant research accomplishments. The service portfolio documents service responsibilities and highlight any service excellence. All portfolios should briefly discuss the educator's philosophy, activities, methods used to implement activities, leadership, mentoring, or committee roles in these respective areas. Recognizing that academic programs have differing needs, this article will attempt to provide some basic guidelines that may help junior faculty in			activities. Also, the portfolio<br><br>is a place to explain why your accomplishments and activities<br><br>are important to the university or to your specialty.<br><br>Each portfolio should be periodically reviewed to assure<br><br>that it reflects the changes occurring in your career. It is a<br><br>living document and should emphasize different things at each<br><br>stage of your career. Finally, it is important to remember that

						diagnostic radiology develop their teaching, research, and service portfolios.			the portfolio is a highly individualized document that should<br><br>reflect your own style
33	University of California San Francisco	-	Educator’s Portfolio	-	-	-	-	Executive Summary<br><br>Teaching<br><br>Mentoring and Advising<br><br>Curriculum Development, Instructional Design and Technology<br><br>Educational Leadership<br><br>Learner Assessment	-
34	Constance D. Baldwin	2010	The Educator Portfolio: A Tool for Career Development	-	-	-	-	Essential Elements:<br><br>Educational Philosophy Statement<br><br>5-year Goals as an Educator	-

								Educational contributors in any or all of 5 categories:<br>Teaching<br>Learner Assessment<br>Curriculum Development<br>Mentoring and Advising<br>Educational Leardership and Administration	
35	Constance Baldwin	-	INSTRUCTIONS FOR EDUCATOR PORTFOLIO TEMPLATE	-	-	-	-	Teaching Assessment of Learners Curriculum Development Mentoring & Advising Educational Leadership & Administration Products of Educational Scholarships	-
36	Uniformed Services University	-	Educator's Portfolio	-	-	-	-	Course Leadership Course Participation Student Mentoring	-

								Supplementals - additional course materials - curriculum development	
37	University of Miami Miller School of Medicine	-	Guidelines for Construction of an Educator’s Portfolio	-	-	-	-	Teaching & Learning Philosophy Mastery of Subject & Personal Development Current Teaching Responsibilities Teaching Performance Mentoring and Advising Curriculum Development & Leadership Instructional Innovation Educational Administration Educational Research & Scholarship	-
38	Academic Pediatrics Association for the PAS Educational	2008	Medical Education Portfolio	-	-	-	-	5-Year Goals as an Educator Description of your Educational Contributions - teaching activities -	-

	Scholars Program							assessment of learner outcomes - evaluation of your teaching - curriculum development - mentoring/advising - educational leadership/ administration - other information (eg. Reviewers, Educational grants, Awards, Publications etc)	
39	Uniformed Services University	-	Sample Faculty Statements	-	-	-	-	Teaching/ educational activities<br><br>Curriculum Development<br><br>Assessment<br><br>Educational Administration and leadership<br><br>Mentoring and advising	-
40	Klara K. Papp	-	Scholarship in Teaching Portfolio	-	-	-	-	Philosophy of Teaching/ Personal Development 5-year	-

								Goals as an Educator Teaching Mentoring and Advising Educational Leadership and Administration Products of Educational Scholarship	
41	Case Western Reserve University	-	Educator Portfolio	-	-	-	-	Philosophy of Teaching/ Personal Development<br>5-year Goals as an Educator<br>Teaching<br>Mentoring and Advising<br>Educational Leadership and Administration<br>Products of Educational Scholarship	-
42	University of Washington	-	The Clinician-Educator's Portfolio: a	-	-	-	-	Executive Summary (1-page maximum)	-

			Template to Document Your Scholarship in Teaching					and Table of Contents<br><br>Personal Information<br><br>a. Name and contact information:<br><br>b. Current position held (academic rank):<br><br>c. Subject area, specialties:<br><br>d. Institutional affiliation:<br><br>e. Time course (years) of activities included:<br><br>f. Short work history<br><br>Teaching Philosophy (1-page maximum)<br><br>Teaching Activities and role as an Educator<br><br>Professional Development in Education	

								Regional/ National/ International Recognition	
43	University of Texas Medical Branch,	-	Educator Portfolio	-	-	-	-	Philosophy and Preparation for Teaching and Educational Leadership<br>Direct Teaching, Mentoring, Advising, and Assessment Activities<br>Educational Materials: Curriculum Development, Dissemination and Scholarship<br>Educational Leadership	-
44	University of Virginia	-	UVa SOM Teaching Portfolio	-	-	-	-	Introductory Statement<br>Contributions as an Educator<br>Teaching Awards	-

								Curriculum and Material Development<br><br>Learner Assessment<br><br>Educational Scholarship/ Creation of Enduring Educational Materials<br><br>Educational Administration and Leadership<br><br>Professional Development in Education<br><br>Mentorship and Guidance	
45	University of Arizona College of Medicine	-	EDUCATOR PORTFOLIO TEMPLATE	-	-	-	-	Educational Philosophy Statement 5-Year Goals as an Educator Revision of your Philosophy and Goals overtime Domains of an Educator Portfolio - teaching - assessment	-

								of learners - curriculum development - mentoring and advising - educational leadership & administration - scholarly approach to education - products of educational scholarship	
46	Weill Cornell Medicine	-	Educator's Portfolio	-	-	-	-	Personal Statement<br>Teaching Activities<br>Teaching Evaluations<br>Development of Curriculum and/or Instructional Materials<br>Development of Assessment Tools of Learner Performance<br>Advising and Mentoring<br>Educational Leadership Roles	-

								Educational Scholarship<br><br>Professional Educational Development<br><br>Educational Honours and Awards	
47	Duke-NUS Medical School	-	EDUCATION PORTFOLIO	-	-	-	-	Executive Summary<br><br>Personal Goals & Values<br><br>Teaching and Supporting Learners<br><br>Educational Research & Evidenced Based Practice<br><br>Educational Management and Leadership	-
48	University of Arkansas for Medical Sciences	-	Educator's Portfolio	-	-	-	-	Direct Teaching Responsibilities<br><br>Curriculum Development	-

								Educational Administration and Leadership<br><br>Assessment of Learner Performance<br><br>Advising and Mentorship	
49	Vanderbilt University Medical Center	-	Educator Portfolio User Guide	-	-	-	-	Teaching Activities<br><br>Teaching Evaluation<br><br>Teaching Awards<br><br>Mentoring and Advising<br><br>Curriculum Development<br><br>Educational Leadership and Administration<br><br>Scholarly Approach to Education<br><br>Products of Educational Scholarship	-

50	John A. Flynn	-	The Educator's Portfolio	-	-	-	-	Evidence of Quantity of teaching effort<br><br>Evidence of Impact<br><br>Evidence of Scholarship (2 types)<br><br>- scholarly teaching: using best practice<br><br>- teaching scholarship: discovering best practice<br><br>Philosophy Statement<br><br>Teaching Activity Report<br><br>Curriculum Development<br><br>Assessment of Learner Performance<br><br>Advisor and Mentor<br><br>Education and Administration<br><br>Public Expression of Scholarly Activity	-

51	Emory University School of Medicine	-	TEACHING PORTFOLIO FOR FACULTY APPOINTMENTS AND PROMOTIONS	-	-	-	-	Description of Teaching Activities<br><br>Department's Assessment of Teaching<br><br>Educational Activities in Professional Organisations<br><br>Awards in Teaching & Biomedical Education<br><br>Letters from Internal/External Reviewers<br><br>Teaching Materials Prepared/Edited<br><br>Public Service Activities<br><br>List of Masters, Doctoral, Post-Doctoral Trainees Supervised<br><br>List of Courses & Self-Instructional Activities Taken to	-

								Improve Teaching Skills	
52	Northwestern University Feinberg School of Medicine,	-	Educator Portfolio	-	-	-	-	Direct Teaching Responsibilities<br>Curriculum Development<br>Advising and Mentorship<br>Educational Leadership<br>Educational Scholarship	-
53	Vanderbilt University Medical Center	-	Teaching Portfolios	-	-	-	-	Thoughts about Teaching Documentation of Teaching Teaching Effectiveness Materials Demonstrating Student Learning Activities to IMprove Instruction Contributions to the Teaching Profession and Institution	-

								Honors, Awards, Recognitions	
54	New York University Langone Health	-	Faculty Teaching Portfolio	-	-	-	-	Personal Statement and Long Term Goals Teaching Effort Mentoring and Advising Administration and Leadership Committees and Teaching Awards Optional Supporting Evidence	-
55	Marshall University, Joan C. Edwards School of Medicine	-	Academic Portfolio: Documenting Teaching, Research, Clinical Care & Service	-	-	-	-	Personal Information<br>Teaching Activities<br>Currciulum / Program Development<br>Research<br>Clinic and Operation<br>Advising and Mentoring<br>Administration and Leadership	-

								Professional Development Activity<br><br>Scholarship<br><br>Honors, Awards Recognitions, and Service<br><br>Outcomes Summary	
56	University of Maryland School of Medicine	-	Teaching Portfolio Guidelines	-	-	-	-	Documentation of Teaching Roles, Activity, Impact/Outcome<br><br>- direct teaching<br><br>- advising & mentoring<br><br>Educational leadership & scholarship<br><br>Teaching Evaluations<br><br>- letters from Students, Graduate Trainees, or Faculty<br><br>Resources	-

57	University of Iowa Health Care	-	Developing a Teaching Portfolio	-	-	-	-	Philosophy of Education<br><br>Curriculum Development<br><br>Teaching Skills<br><br>Assessment of Learner Performance<br><br>Advising<br><br>Educational Administration<br><br>Regional/National Scholarship<br><br>Continuing Education<br><br>Teaching Awards<br><br>Long-Term Goals	-
58	Lee A. Learman	-	The Educator Portfolio: An Essential Component of Your Promotion Dossier	-	-	-	-	Educational Philosophy Statement<br><br>5-Year Goals as an Educator	-

								Revision of your Philosophy and Goals overtime<br><br>Domains of an Educator Portfolio<br><br>- teaching<br><br>- assessment of learners<br><br>- curriculum development<br><br>- mentoring and advising<br><br>- educational leadership & administration	
59	Upstate Medical University College of Medicine	-	Educator Portfolio Checklist and Tips	-	-	-	-	Educational Philosophy Statement - role as teacher - theory of learning - characteristics of good teachers - role/responsibility of learners 5-year Goals as an educator Documentation of	-

								educational contributions in any of the following 5 categories: - teaching - learner assessment - curriculum development - mentoring and advising - educational leadership and administration	
60	University of Florida College of Medicine	-	Portfolio for Applicants to the Society of Teaching Scholars	-	-	-	-	Education Narrative Excellence in Teaching Excellence in Educational Scholarship Self-Improvement, Educational Leadership and Service Contributing to Academic Excellence by Mentoring	-
61	Washington University School of	-	CLINICAL EDUCATOR PORTFOLIO	-	-	-	-	Clinical Contributions Educational Contirbutions - direct teaching -	-

	Medicine in St. Louis		TEMPLATE GUIDELINES					educational leadership - currculum development - development of new methods of education - mentorship provided - development/ participation in CME courses - feedback - literature Community Service Contributions	
62	University of Colorado School of Medicine	-	Membership - Academy of Medical Educators	-	-	-	-	Direct Teaching<br>Advising & Mentorship<br>Instructional development and Curricular Design<br>Educational Administration and Leadership<br>Educational Scholarship and Research	-

63	McMaster University Health Sciences	-	Teaching Portfolio	-	-	-	-	Executive Summary<br>Supporting Documentation<br>- educational contributions<br>- peer review and letters of support from colleagues<br>- performance reviews and letters of appreciation from educational administrators<br>- course evaluations<br>- external materials	-
64	Thomas Jefferson University	-	JEFFERSON TEACHING PORTFOLIO	-	-	-	-	Direct Contributions to Teaching<br>- teaching activities<br>- curriculum design or development<br>- instructional materials	-

								Educational Adminsitration/ Committee Service<br>- leadership & membership roles<br>- professional reviewer/moderator activities related to education at national level<br>Educational Scholarship<br>Mentoring<br>- mentoring and advising<br>Teaching Awards/Honors or Other Recognition for Educational Contributions<br>Faculty Development<br>Reflective Statement	
65	Lingnan University	2015	An Introduction to "The Full Teaching	-	-	-	-	Summary of Teaching Responsibiltiies	-

			Portfolio" A Guide for Academic Staff					Reflective Statement on Teaching Philosophy, Practices, and Goals Course Development and Modification Development of Teaching Materials Products of Good Teaching Steps taken to Evaluate & Improve Teaching Presentations, Research, and Publications on Teaching Administrative & Committee Work Related Teaching Information from Students Information from Colleagues Information from other Sources	
66	Sandro Pinheiro	-	Documenting Success: Documenting Success: Developing The Educator	-	-	-	-	Summary of Teaching Activities<br>Curriculum Development and	-

			Developing The Educator's Portfolio					Instructional Materials<br>Educational Administration and Leadership<br>Educational Scholarship<br>Awards and Honors<br>Professional Development in Education<br>Intellectual-Reflective Statement/ Philosophy of Education	
67	Ankeet Udani	-	My Educator Portfolio	-	-	-	-	Philosophy Development Activities Leadership Scholarship Awards Advising Mentors Goals	-
68	Donna M. D'Alessandro	-	Medical Educator's Portfolio	-	-	-	-	Teaching<br>Dissemination/ Scholarship of Teaching	-

								Personal Statement	
69	University of Rochester School of Medicine & Dentistry,	-	The Educator Portfolio: Sample Template	-	-	-	-	Statement of Educational Philosophy<br>Description of Educational Contributions:<br>- teaching<br>- assessment of learners<br>- curriculum development<br>- mentoring and advising<br>- educational leadership and administration	-
70	Medical University of South Carolina	-	Academy of Medical Educators	-	-	-	-	Personal Statement<br>CV<br>Record of Achievement in Multiple Educational Doamins	-

71	University of Western Ontario	-	The Teaching Dossier	-	-	-	-	Teaching Responsibilities Scholarship & Research on Teaching Evidence of Excellence Educational Administration and Leadership	-
72	Association of Pulmonary and Critical Care	-	The Educator Portfolio: A Tool for Reflection and Personal Development	-	-	-	-	Thoughts about Teaching Educational Contributions Recognition of Excellence Activities to improve expertise as educator Contribution to profession & scholarship	-
73	MGH Institute of Health Professions	-	How do you create a teaching portfolio?	-	-	-	-	Teaching Responsibilities Reflection on teaching goals, philosophy & style Teaching Philosophy Statement Best examples of work, Connecting to	-

								teaching philosophy share and review portfolio with colleague	
74	Dawn Bowdish	-	Teaching Portfolio	-	-	-	-	Description of Responsibilities Teaching Approach/Philosophy Teaching Practice Contributions to Teaching Responses to the Summative Question in students' ratings	-
75	National Health Service	-	E-portfolios for doctors	-	-	-	-	Teaching Sessions with Feedback Received Certificaties of Courses Degrees Diplomas Prizes Other relevant courses Documents used to record Foundation Competencies Audits, Presentations, Publications (include reflections &	-

								feedback) Emails of impending audits and publications Feedback, Thank you letters of praise from colleagues/ patients achievements from medical school	
76	Linda Drumheller	-	Leadership Careers in Medical Education	-	-	-	-	Direct Teaching<br><br>Assessment of Learner Performance<br><br>Mentoring/ Advising<br><br>Curriculum Development<br><br>Educational Administration and Leadership	-
77	Peter Seldin	-	The Teaching Portfolio: A Practical Guide to Improved Performance and Promotion/Tenure Decisions	-	-	-	-	Personal Narrative<br><br>Teaching Philosophy<br><br>Teaching Responsibilities<br><br>Evidence of Successful Innovations	-

								Accomplishments & Awards<br><br>Professional Goals	
78	Association of University Radiologists	-	Clinical Faculty Track - Portfolio Requirements	-	-	-	-	CV Most Current Faculty Evaluation Recommendation Reports/Letters Summary of Service Activities Evidence of Teaching Effectiveness Evidence of Scholarship Calendar for Submission of Promotion Award Proposals	-
79	International Association of Medical Science Educators,	-	CAMSE Educator Sample	-	-	-	-	Teaching Learner Assessment Curriculum Development Advising and Mentoring Educational leadership and Administration	-
