# Supplementary material for: A Systematic Scoping Review on Portfolios of Medical Educators
Source: J Med Educ Curric Dev. 2021 Mar 24;8:23821205211000356. doi: 10.1177/23821205211000356 (PMC8855455; doi:10.1177/23821205211000356)
Supplement: sj-pdf-5-mde-10.1177_23821205211000356 – Supplemental material for A Systematic Scoping Review on Portfolios of Medical Educators [file sj-pdf-5-mde-10.1177_23821205211000356.pdf]

MEDICAL EDUCATION PORTFOLIO TEMPLATE

1. PERSONAL PARTICULARS

Name:
Home Phone:
Office Phone:
Address:
Current Academic Rank:

2. PERSONAL STATEMENT

- Teaching Philosophy
  For example: your approach to education, principles that underlie your teaching

- Personal & Professional Goals as an Educator
  For example: list of aspirations for achievement, plans for learning and growth

3. TEACHING AND SCHOLARSHIP

- Teaching Activities
  Describe the quantity and quality of your teaching activities
  List important teaching activities – eg. Lectures, workshops
  Reflect on your planning process and its impact on learners

- Educational Scholarships
  List your educational scholarships

4. MENTORING AND ADVISING

- List of mentees and their progressions
  Reflect on how mentoring/advising experience has added value to you as a medical educator

Name of Mentee/Advisee	Dates	Mentee's position/role at start of mentoring	Mentoring Focus (eg. Research)	Outcomes: Mentee's current position and achievements

5. EDUCATIONAL RESEARCH PRODUCTS

- List of research papers and other educational research products

6. LEADERSHIP AND ADMINISTRATION

- Educational Leadership and Administration Descriptions
  List your past or present leadership roles in education – eg. List your roles, level, dates and outcomes in programs, courses, educational committees
  Reflect on your experiences in these roles

Role in Program/course/educational committee	Level	Dates	Outcomes

7. CURRICULUM DEVELOPMENT

- Description of curriculum development activities

Curriculum Topic	Type of Learners	Was it implemented?	Where it was implemented	Curriculum Description

8. LEARNER ASSESSMENT

- Description of learner assessment methods employed/deployed

Assessment Method	Context of Assessment	Assessment Role	No. and types of Learners assessed	Outcomes of Teaching Effectiveness

9. FORMAL RECOGNITION

- List any formal recognitions

10. PROFESSIONAL DEVELOPMENT

- Describe your professional developments in medical education
- List any professional development activities
